# Evolutionary and Transmission Dynamics of Reassortant H5N1 Influenza Virus in Indonesia

**DOI:** 10.1371/journal.ppat.1000130

**Published:** 2008-08-22

**Authors:** Tommy Tsan-Yuk Lam, Chung-Chau Hon, Oliver G. Pybus, Sergei L. Kosakovsky Pond, Raymond Tze-Yeung Wong, Chi-Wai Yip, Fanya Zeng, Frederick Chi-Ching Leung

**Affiliations:** 1 School of Biological Sciences, The University of Hong Kong, Hong Kong Special Administrative Region, China; 2 Department of Zoology, University of Oxford, Oxford, United Kingdom; 3 Department of Pathology, University of California San Diego, La Jolla, California, United States of America; The Pennsylvania State University, United States of America

## Abstract

H5N1 highly pathogenic avian influenza (HPAI) viruses have seriously affected the Asian poultry industry since their recurrence in 2003. The viruses pose a threat of emergence of a global pandemic influenza through point mutation or reassortment leading to a strain that can effectively transmit among humans. In this study, we present phylogenetic evidences for the interlineage reassortment among H5N1 HPAI viruses isolated from humans, cats, and birds in Indonesia, and identify the potential genetic parents of the reassorted genome segments. Parsimony analyses of viral phylogeography suggest that the reassortant viruses may have originated from greater Jakarta and surroundings, and subsequently spread to other regions in the West Java province. In addition, Bayesian methods were used to elucidate the genetic diversity dynamics of the reassortant strain and one of its genetic parents, which revealed a more rapid initial growth of genetic diversity in the reassortant viruses relative to their genetic parent. These results demonstrate that interlineage exchange of genetic information may play a pivotal role in determining viral genetic diversity in a focal population. Moreover, our study also revealed significantly stronger diversifying selection on the *M1* and *PB2* genes in the lineages preceding and subsequent to the emergence of the reassortant viruses, respectively. We discuss how the corresponding mutations might drive the adaptation and onward transmission of the newly formed reassortant viruses.

## Author Summary

H5N1 highly pathogenic avian influenza (HPAI) virus emerged in China in 1996, and has spread beyond Asia since 2003. Following the first outbreak reported in Indonesian poultry farms in December 2003, the virus spilled over to 27 Indonesian provinces by June 2006, and became endemic in the country. In the following years, repeated sporadic human infections in Indonesia had been attributed to H5N1 HPAI viruses. Nonetheless, the viral evolution and transmission have not been fully understood. Here, we report phylogenetic evidence of a group of interlineage reassortant viruses isolated from human and cats in Java. Our comparative study of the reassortant viruses and one group of genetic parents found that although their rates of evolution were similar and both of their phylogenies were not geographically structured within mainland Java, the growths of genetic diversity were different. We also detected significant positive selection on the viral matrix and polymerase genes preceding and subsequent to the emergence of the reassortant viruses, which might correspond to viral adaptation. Based on our findings, we discuss the possibility of host switching in facilitating the emergence of the reassortant strain, and call for more extensive viral surveillances in the non-avian population in Indonesia.

## Introduction

The H5N1 highly pathogenic avian influenza (HPAI) virus was originally isolated from a farmed goose in Guangdong province of China in 1996 [1], and soon spread to live-poultry markets in Hong Kong [2], resulting in 18 cases of human infection in 1997, 6 of which were fatal [3],[4]. The first wave of H5N1 infection ceased after the depopulation of all poultry in Hong Kong, although the H5N1 virus was later found to circulate continuously in Southern China without causing apparent disease symptoms among infected poultry [5]. H5N1 outbreaks recurred in 2003, persistently affecting poultry farms in many Southeast Asia countries, such as China, Thailand, Vietnam, Indonesia and Cambodia. The viruses also spread outside Asia, including to some European countries. More importantly, occasional zoonotic transmissions to humans occurred in most of the affected Asian countries and the virus continued to pose a serious threat to global public health [6].

H5N1 outbreaks in Indonesia were initially detected in poultry farms in December 2003 [7]. It was suggested that the H5N1 virus was first introduced to Java and subsequently spread to other parts of the country [8]. The virus rapidly became endemic in Indonesia [9],[10], and continued to cause sporadic zoonotic transmissions to humans beginning in July 2005 [9]. Three clusters of H5N1 transmission among family members were identified in 2005, raising concerns of possible human-to-human transmission of the virus [11],[12]. As of April 8, 2008, Indonesia had 132 confirmed human cases with 107 deaths [13], the largest number of deaths among all affected countries.

Previous studies have shown that several H5N1 genotypes have emerged in Asia through reassortment between H5N1 viruses and other subtypes [14],[15]. One of these genotypes, Z, predominated the H5N1 outbreaks throughout 2003–2007, causing most H5N1 outbreaks in Asian countries, including Indonesia [16]. Moreover, a variety of antigenically distinct sublineages of Z genotype virus have been established [16]. Unlike Vietnam and Thailand, Indonesia was invaded by only a single sublineage of genotype Z virus. Previous phylogenetic analyses suggested that Hunan province of China may be the source of the initial H5N1 outbreak in Indonesia [17], and classified the Indonesian H5N1 HPAI viruses into three groups [10]; however, further statistical analysis is necessary to characterize and compare different aspects of their evolutionary histories. In this study, we examined molecular phylogeny of the most recent Indonesian H5N1 viruses isolated from avian and mammal hosts. A group of putative reassortant viruses was discovered and their genetic parents were identified. In addition, we investigated the evolutionary behaviors (including spatial migration, growth of genetic diversity, and evolutionary drift and selection) of the reassortant viruses and compare with those of the parental strain, thereby providing insights into the nature and impact of this emerging reassortant strain.

## Results

### Phylogenetic relationships among Indonesian H5N1 viruses

**Figure 1:**
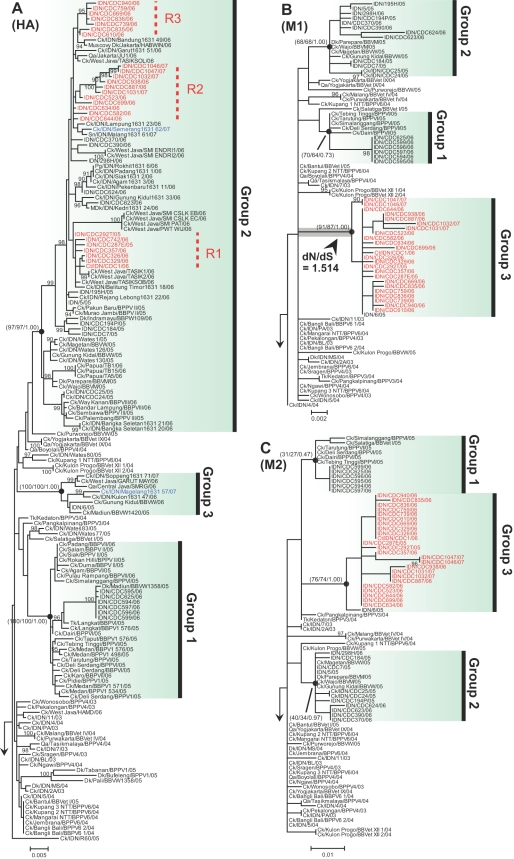
Phylogenetic trees of Indonesian H5N1 influenza viruses. ML phylogenies reconstructed from (A) *HA* gene; (B) *M1* gene; (C) *M2* gene. Topological supports (>90) summarized from 1,000 ML bootstrap replications are shown. For major lineages, NJ bootstrap support (1,000 replications) and posterior probability from BMCMC analyses (5,000 tree samples) are also shown inside parentheses (ML/NJ/BMCMC). Putative human and cat reassortant viruses are in red. Reassortant subgroups (R1, R2, and R3) are further indicated with dashed lines in (A). Putative avian reassortant viruses are in blue. The pre-emergence lineage (refer to main text) is highlighted in gray in *M1* phylogeny (B). Arrows indicate the roots. The distance unit is substitutions/site.

Phylogenetic trees of Indonesian H5N1 viruses were reconstructed from 12 separate gene datasets (Table S6), using a maximum likelihood (ML) approach with bootstrapping analyses to assess clade robustness (Figures 1, S1–S3; computer files of dendrogram are available as Dataset S1). In all the phylogenies, viruses sampled from avian species during earlier years of outbreaks (predominantly 2003–2004) tended to cluster near the root as expected, but with a poorly resolved branching structure that is likely due to relatively low sequence divergence. In contrast, viruses sampled from recent infections (2005–2007) from avian and mammalian hosts formed three well-supported lineages with bootstrap support (or posterior probabilities) over 90 (or 0.9) under neighbor-joining (NJ), ML and Bayesian Markov Chain Monte Carlo (BMCMC) methods. We denote these lineages as groups 1, 2, and 3 in the hemagglutinin (HA) and neuraminidase (NA) phylogenies (Figures 1A and S2C). This structure was preserved in the phylogenies of other genes for which sufficient sequence data were available (viruses from group 3 were missing sequence data for the *NP*, *NS*, *NS1*, *NS2*, and *PB2* genes). The group 3 lineage in the *MP*, *M1*, *M2*, and *PB1* phylogenies was only represented by the A/Indonesia/6/05 strain.

It is important to recognize that our phylogenetic groupings (groups 1, 2, and 3) of Indonesian H5N1 viruses (Figure 1 and Table S6) are slightly different to those by Smith and coworkers [10] who did not require the same level of clustering support for each group, leading to the inclusion of earlier viruses (predominantly 2003–2004). We chose to be conservative, and did not include poorly supported branches (e.g., earlier viruses) in our viral group definition. Therefore, we did not define a group corresponding to the group B of Smith et al., because most of group B taxa are earlier viruses. Groups 1 and 2 in this study correspond to groups C and A defined by Smith et al. respectively, plus some more recent viruses. Smith did not report group 3, because the sequences were unavailable at that time.

### Identification of reassortant viruses

We found previously unrecognized phylogenetic discordance between gene trees involving human and cat isolates (n = 25, denoted in red in Figures 1, S1–S3)—the main focus of our study—suggesting that they are reassortant viruses descending from group 2 and 3 lineages. In addition, the placement of two avian viruses isolates from Java (Ck/IDN/Semerang1631-62/07 and Ck/IDN/Magelang1631-57/07, shown in blue in Figures 1A and S2C) differed between *HA* and *NA* phylogenies, suggesting another reassortment event.

**Figure 2:**
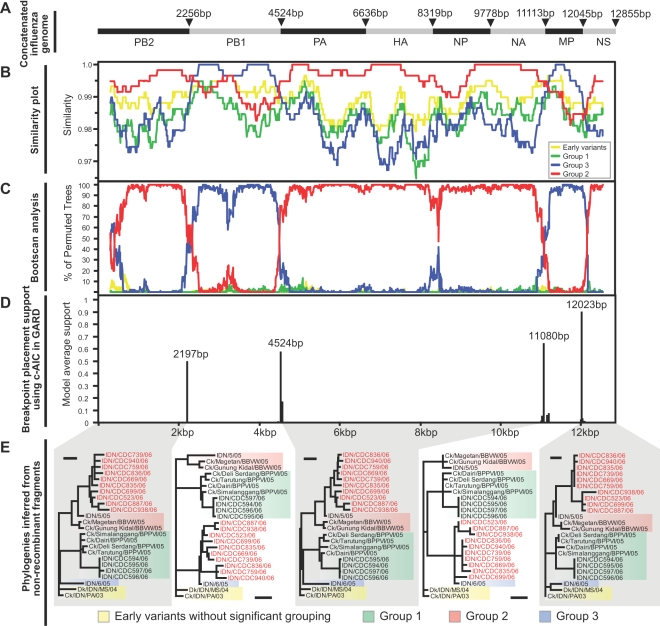
Recombination analyses on concatenated influenza virus genomes. (A) Schematic diagram of concatenated influenza virus genomes. (B) Similarity plot. (C) Bootscan analysis. (D) GARD analysis. (E) Individual phylogenies reconstructed from non-recombinant fragments identified by GARD. Consensus sequences representing viral groups, window size of 600 bp and step size of 10 bp, were used for the similarity plot and bootscan analysis. The distance bar for the trees in (D) is 0.004 substitutions/site. Taxa of putative reassortant viruses are in red.

To further investigate the putative reassortant human and cat viruses, a selected dataset (n = 24) of manually concatenated full genomes (Figure 2A; see Methods) of Indonesian H5N1 HPAI viruses were analyzed using more sophisticated analysis methods, including similarity plots, bootscan analyses and GARD analyses (genetic algorithm for recombination detection). In the similarity and bootscan plots (Figure 2B and 2C), the putative reassortants (represented by a consensus sequence) showed a high degree of sequence similarity and phylogenetic clustering with the group 3 strain A/Indonesia/6/05 in the *MP* and *PB1* segments, but not in other genomic regions, where they were more similar to the consensus sequence of group 2 viruses (Figure 2B and 2C). Moreover, GARD detected two well-supported breakpoints near the boundaries of *MP* and *PB1* segments in the concatenated genomes (Figure 2D), suggesting that the phylogenetic incongruence was significant between the three regions. In summary, all three analyses agreed that the newly reassortant strains had arisen from acquiring *PB1* and *MP* genome segments from the group 3 lineage and the remaining segments from the group 2 lineage.

### Genetic, temporal, and geographical origin of reassortant viruses

Based on the HA phylogeny (Figure 1A), we further classified the reassortant viruses into three subgroups (R1, R2, and R3) with bootstrap support of 80% or better, as shown in the phylogenies containing only reassortant viruses (Figure S4). Similar groupings were observed in the NA phylogeny (Figures S2C and S4B), although here subgroup R3 clustered with subgroup R1, and two reassortant viruses isolated in 2007 (IDN/CDC1046/07, IDN/CDC1047/07) moved to a different subgroup. These inferred clustering patterns can be explained by multiple reassortment events, or by a single reassortment followed by divergence due to mutation and selection in different populations. We note that some group 2 viruses also cluster inside the reassortant subgroups (Figures 1A and S2C) and may indicate more reassortment events; however, most of them formed polytomies close to the most recent common ancestor (MRCA) of the reassortant subgroups and had poor bootstrap support for their exact placement. As the divergence between the reassortant subgroups and other intercalating group 2 viruses are low, the three subgroups may actually be linked uninterruptedly, implying a single origin. Therefore, the times and number of reassortment events that generated the putative mosaic reassortant viruses remains elusive. We examine both the single and multiple origin hypotheses in subsequent analyses, excluding the intercalating group 2 viruses from the reassortant group.

**Figure 3:**
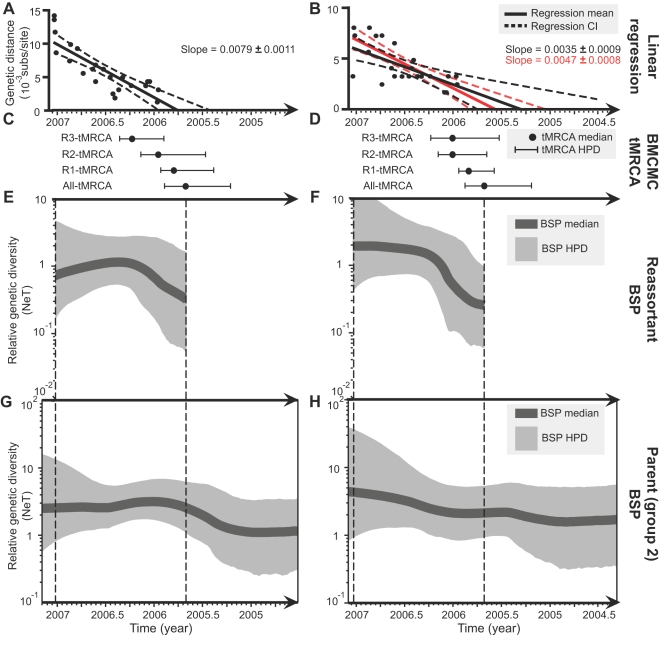
Estimation of tMRCA and relative genetic diversity for reassortant viruses and its genetic parent (group 2). (A) and (B) show the plots of the genetic distance from MRCA to each reassortant taxa, and the linear regression line depicting the tMRCA for *HA* and *NA* genes, respectively. 95% confidence intervals are shown by dashed lines. Red dots and regression line indicate the removal of two sequences from the *NA* dataset. (C) and (D) show the tMRCAs estimated respectively from *HA* and *NA* genes using the BMCMC method. 95% higher probability density (HPD) is shown by the error bar. (E) and (F) show the Bayesian Skyline plots (BSP) illustrating the change of relative genetic diversity of reassortant viruses through time estimated from the *HA* and *NA* gene datasets, respectively. (G) and (H) show the BSP for group 2 viruses estimated from *HA* and *NA* gene datasets, respectively.

To estimate the times of the reassortment events that generated the putative reassortant viruses, the times of the MRCA (tMRCA) of the three reassortant subgroups were estimated using BMCMC methods [18],[19]. Bayes Factors (BF) [20] were used to select among strict and relaxed clock models of evolution [21]. The uncorrelated exponentially-distributed clock model (UCED) significantly outperformed the other models (lnBF>3) for most datasets, except for the NA gene of the reassortant viruses, for which the strict clock model was not rejected (lnBF<1; Table S4). The results of tMRCA estimation are summarized in Figure 3C and 3D. In addition, sequence isolation dates were plotted against their genetic distance (units of substitutions/site) to their MRCA, to graphically show the accumulation of mutations through time (Figure 3A and 3B). The tMRCA of all reassortant viruses (All-tMRCA) was dated to July 2005 (highest probability density, HPD confidence interval: April–October), which is consistent with the linear regression estimate in Figure 3A and 3B. However, the regression estimate of All-tMRCA for the *NA* gene (April 2005) is slightly older than BMCMC estimate (July 2005). If strains IDN/CDC1046/07 and IDN/CDC1047/07 are excluded from regression analyses then the All-tMRCA date (July 2005; as indicated by the red regression line in Figure 3B) becomes consistent with the BMCMC estimate (July 2005). This suggests that the regression method was sensitive to rate variation caused by the two potential recent reassortants. The tMRCA estimates (R1-tMRCA, R2-tMRCA, and R3-tMRCA) for the individual reassortant subgroups range from May 2005 to April 2006 (Figure 3C and 3D).

**Figure 4:**
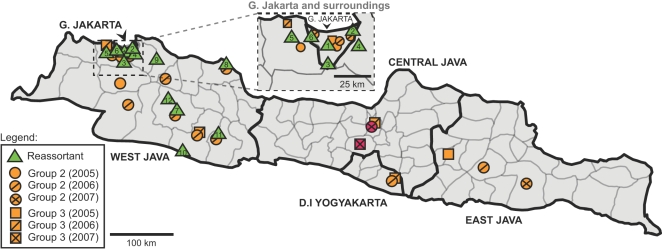
Map of mainland Java in Indonesia. Locations for the putative reassortant viruses focused in this study are indicated with green triangles. Numbers inside the triangles refer to the locations of the reassortant viruses described in Table 1. Locations of group 2 and 3 viruses are indicated with orange circles and squares, respectively. Red circles and squares denote the locations of putative avian reassortant viruses in 2007. Only those parental strains genetically close to the reassortant viruses are shown in the map. A zoom-in of Greater Jakarta (G. Jakarta) and surroundings is illustrated separately in a dashed-line bounded box in the centre of the figure.

The majority (17/25) of reassortant viruses were isolated from Greater Jakarta and surrounding areas such as Bekasi and Banten (Table 1 and Figure 4). The remaining eight samples were isolated from more distant locations in West Java province, such as Indramayu and Karawang. The two earliest reassortant viruses were isolated from central and east Jakarta (Table 1). Parsimony reconstruction (see Methods) of binary ancestral geographical states (either Greater Jakarta or West Java) upon the *HA* and *NA* ML phylogenies suggested that the MRCA of all reassortants (and the MRCAs of each reassortant subgroup) likely originated from Greater Jakarta and surroundings (Table S2; result robust to random resolution of polytomies; see Methods).

**Table 1:** Cases of human infections caused by Indonesian reassortant H5N1 HPAI viruses.

Sub- group	Strain name	Case index	Fatal	Sex	Age	Family cluster	Place^f^	Onset date	Death date	Sampling date
R1	IDN/CDC292/05	15	Y	M	8		Central Jakarta (1)	8-12-05	15-12-05	15-12-05
R1	IDN/CDC742/06	56	Y	F	17		Jakarta (1)	28-7-06	8-8-06	7-8-06
R1	IDN/CDC287/05	16	Y	M	39		East Jakarta (2)	9-12-05	12-12-05	13-12-05
R1	IDN/CDC357/06	21	Y	M	15		Padalarang, Bandung, West Java (12)	n/a	1-2-06	30-1-06
R1	IDN/CDC326/06	19	Y	M	4	E^c^, brother	Indramayu, West Java (8)	8-1-06	17-1-06	15-1-06
R1	IDN/CDC329/06	18	Y	F	13	E^c^, sister	Indramayu, West Java (8)	6-1-06	14-1-06	14-1-06
R3	IDN/CDC940/06	74	Y	M	30 m		Karawang, West Java (9)	5-11-06	13-11-06	12-11-06
R3	IDN/CDC759/06	59	Y	F	35		Cikelet, West Java (10)	8-8-06	17-8-06	17-8-06
R3	IDN/CDC669/06	51	Y	M	13		South Jakarta (3)	9-6-06	14-6-06	13-6-06
R3	IDN/CDC836/06	68	Y	M	20		Bandung, West Java (7)	17-9-06	28-9-06	24-9-06
R3	IDN/CDC739/06	55	Y	M	16		Bekasi, West Java (4)	26-7-06	7-8-06	5-8-06
R3	IDN/CDC835/06	67	Y	M	9		South Jakarta (3)	13-9-06	22-9-06	22-9-06
R3	IDN/CDC610/06	41	Y	M	12		Bekasi, West Java (4)^a^	7-5-06	13-5-06	11-5-06
R2	IDN/CDC644/06	49	Y	M	15		Tasikmalaya, West Java (11)	24-5-06	30-5-06	30-5-06
R2	IDN/CDC1046/07	77	Y	F	22		Tangerang, Banten (5)	3-1-07	12-1-07	11-1-07
R2	IDN/CDC1047/07	78	Y	F	27		South Jakarta (3)	6-1-07	12-1-07	12-1-07
R2	IDN/CDC1032/07	76	Y	F	37	M^d^, mother	Tangerang, Banten (5)	1-1-07	11-1-07	6-1-07
R2	IDN/CDC938/06	73	Y	F	35		Tangerang, Banten (5)	7-11-06	28-11-06	10-11-06
R2	IDN/CDC887/06	71	Y	M	11		South Jakarta (3)	2-10-06	14-10-06	14-10-06
R2	IDN/CDC1031/07	75	Y	M	14		West Jakarta (6)	31-12-06	10-1-07	5-1-07
R2	IDN/CDC523/06	30	Y	F	10 m		Kapuk, West Jakarta (6)	17-3-06	23-3-06	23-3-06
R2	IDN/CDC699/06	53	Y	F	3		suburb of Jakarta (5)^b^	23-6-06	6-7-06	6-7-06
R2	IDN/CDC634/06	44	Y	F	10	H^e^, sister	Bandung, West Java (7)	16-5-06	23-5-06	23-5-06
R2	IDN/CDC582/06	33	Y	M	30		Jakarta (1)	17-4-06	26-4-06	26-4-06

a Some information sources refer to this as East Jakarta.

b Some information sources refer to this as Tangerang.

c There are two other family members (father and sister) suspected with H5N1 infections (non-fatal).

d There is another family member (son) who was confirmed with H5N1 infection (index #79, non-fatal); however, virus sequences are not available.

e There is another family member (brother) who was confirmed with H5N1 infection (index #45, fatal); however, virus sequences are not available.

f Numbers in parentheses are the unique references to the localities shown in Figure 4. Numbers 1–6 were assigned to Greater Jakarta and surroundings; numbers 7–12 were assigned to West Java.

n/a = Information not available.

### Spatial migration of reassortant and its parental strain

The mean numbers of observed geographical state changes (GSC) of the reassortant and of the group 2 parental strains were estimated independently and compared with the null distribution of GSC values under the null hypothesis of completely unrestricted migration (i.e. panmixis; Figure S5) [22]. For the reassortant strain, the observed GSC value was not significantly lower than the GSC value expected under panmixis (Slatkin-Maddison test: *p*>0.2). Therefore the observed geographic structure is not significantly different to that expected by chance alone. For group 2 viruses, the observed GSC value for all geographical state pairs is significantly (*p*<0.0002) lower than the null value. However, the observed value of GSC within Java (i.e., migrations between Greater Jakarta and the rest of Java) and between the three islands (i.e. migrations between mainland Java, Sumatera and Sulawesi Selatan including Papua) are insignificantly (*p*>0.2) and significantly (*p*<0.0002) lower than the corresponding null values respectively, suggesting that the phylogeny of group 2 viruses is not geographically structured within Java, but is subdivided by island-to-island migrations. However, we could not address whether the viral migrations inside Sumatera and Sulawesi Selatan including Papua are panmictic or structured due to limited operative localities in our dataset to distinguish between different regions inside these islands. We also found that the migration of group 2 viruses from Greater Jakarta and surroundings to Sumatera and Sulawesi Selatan including Papua was more frequent than expected under the null hypothesis, and there is relatively little viral migration from the rest of Java to Sumatera and Sulawesi Selatan including Papua (Table S3). This observation suggests Greater Jakarta played a more salient role in dispersing group 2 viruses to other Indonesian islands than other parts of Java did.

### Population dynamics of reassortant and its parental strain

We used the Bayesian skyline plot (BSP) [23] to estimate the change of relative genetic diversity of the reassortant viruses and of the group 2 parental strain over time, as shown in Figure 3E–3H. For both the *HA* and *NA* datasets, the group 2 viruses consistently show a slow growth in relative genetic diversity over time which appears to follow a constant size or exponential growth model, whereas the reassortant viruses initially exhibited an abrupt rise in relative genetic diversity followed by stabilization, which visually resembles a logistic growth curve with two phases [24],[25] (Figure 3E and 3F). When the BSPs are superimposed upon the demographic results obtained under parametric growth models (i.e., constant, exponential and logistic growth; Figure S8), then a similar observation can also be made. However, BF tests (Table S4) indicate there is insufficient statistical power to discriminate between the three parametric growth models (lnBF<2.99), suggesting a lack of strong demographic signal in these data. When the parametric demographic models were fitted to the data, the median estimates of growth rates for the reassortant datasets are generally higher than those estimated for the datasets of group 2 viruses (Table S1). However, the confidence intervals of some growth rate estimates are fairly large and overlapped among the reassortant and group 2 viral datasets.

### Diversifying selection in the *PB2* and *M1* genes

Using the Random Effects Likelihood (REL) method [26] we found sites under positive selection in the *PB2* gene (codons 76, 534, 627, 677 and 740) and the PA gene (codon 409) of the reassortant viruses. The Fixed Effects Likelihood (FEL) method [26] was more conservative and only identified *PB2* codon 534 as being positively selected. For the group 2 viruses, *HA* codon 129 (starting from HA1) and *M1* gene codon 205 were the only selected sites identified by the FEL and REL methods, respectively. Using a lineage-specific selection model (see Methods), we identified elevated rates of diversifying selection, measured by the ratio of non-synonymous to synonymous substitutions (dN/dS), on the *M1* gene in the lineage leading to the MRCA of the group 3 viruses and preceding the emergence of the reassortant viruses (highlighted in Figure 1B). The dN/dS values for the *M1* gene in this lineage (which we call the pre-emergence lineage) was estimated to be 1.514 (95% CI: 0.447–3.814; see Table S5), significantly higher (LRT *p*<0.002) than the mean estimates for other lineages (dN/dS = 0.077) in the Indonesian clade and for lineages in other H5N1 HPAI clades (e.g., Fujian, Qinghai, Thailand and Vietnam clades which have dN/dS ranging from 0.05 to 0.09). This lineage-specific elevation of dN/dS was not significant (LRT *p*>0.1) for other genes (i.e. *HA*, *NA*, *M2*, *PB1*; see Table S5). Four amino acid changes in *M1* occurred along the pre-emergence lineage, including threonine to alanine at reside 37, arginine to lysine at reside 95, threonine to alanine at reside 137, glutamine to histidine at reside 249. Three (residue 37, 95, and 137) of them are located close to the electrostatic positive surface of the N-terminal domain of the M1 protein molecule (Figure S7), and one (residue 249) is located in the remaining C-terminal fragment.

## Discussion

This study classified H5N1 HPAI viruses in Indonesia into three distinct viral lineages (groups 1, 2, and 3) and discovered a group of naturally occurring reassortant viruses that represent a newly emergent H5N1 HPAI strain in Java in 2006. Several phylogenetic methods concurred that two (*MP* and *PB1*) of the reassortant viruses' genome segments descended from the group 3 ancestral viruses, and the remaining six (*PB2*, *PA*, *HA*, *NP*, *NA*, *NS*) segments descended from the group 2 ancestral viruses. Although the majority of reassortant viruses (24/25) are human isolates, few of the associated human infections are epidemiologically linked (Table 1), suggesting multiple sporadic zoonotic transmissions from birds. The phylogeographic results indicate that the parental viruses of the reassortants have been co-circulating in Java since 2005. Despite the identification of parental lineages, the exact number of reassortment events remains difficult to assess. Although the three fairly consistent phylogenetic subgroups (subgroups R1, R2, and R3 in Figure S4) formed by the reassortant viruses suggest three independent reassortments, the underlying uncertainty in our estimated phylogenies means that we cannot rule out the possibility of a single origin.

The hypothesis of three reassortments implies that the viruses have acquired exactly the same genome segments from the same group of parental viruses, which seems unlikely to occur by chance (probability = 0.0089, assuming panmixis and that exactly two genomic segments are swapped out). This probability might be increased if reassortments confer a selective advantage. We did detect a significantly stronger selection pressure on the M1 protein in the pre-emergence lineage of group 3 parental strain that led to the reassortant viruses (Table S5). Previous reports suggested a few amino acid changes in M1 of influenza A and B viruses can confer a growth advantage in mouse lungs [27]–[29]. Although the M1 mutations identified in this pre-emergence lineage have not been functional characterized elsewhere in the authors' knowledge, one (residue 137, T→A) of them is close to a previously characterized mutation (residue 139, T→A) which controls the virulence in mouse model [27],[29]. Three of the inferred residue changes are located close to the electropositive surface of N-terminal domain of M1 protein (Figure S7) that acts to bind viral RNA [30],[31]. The M1 matrix protein mediates encapsulation of viral RNA-nucleoproteins into membrane envelope during packaging [31], and has close contact with other viral proteins inside the viral particle. It seems possible that some of these changes may be involved in the adaptation of reassortant viruses, through promotion of structural interactions among viral proteins.

According to our analyses, the common ancestor of the reassortant viruses is dated to July 2005 (HPD: April–October), approximately 5 months prior to the first case of human infection caused by the reassortant virus (index case #15 defined by WHO; see Table 1). Our analysis of virus phylogeography suggests the ancestors of these reassortant viruses first arose in Greater Jakarta and surroundings, which agrees with the observation that the first two cases of human infection by the reassortant viruses occurred in Central and East Jakarta (index cases #15 and #16). The molecular dating and phylogenetic analyses suggest that nascent reassortant viruses might take several months to spread and expand their diversity in the local bird population, eventually leading to the exposure of human population. The subsequent spread of the reassortant strain seems to become more rapid and extensive, as human cases were reported outside Greater Jakarta one month later, and the reassortant virus spread to as far as the south and east of West Java in the following six months (Table 1 and Figure 4). Commercial poultry transportation, as well as carriage by migratory birds, may facilitate the viral migration, but their tangible contributions need further studies. Our results suggest that the circulations of reassortant viruses and their genetic parent (group 2) were not restricted by geography within Java. The viral migration back to Greater Jakarta could be driven by the inter-province transfer of infected poultry, in particular the importation of live poultry or fresh poultry products to the densely human populated Jakarta from the remote provinces engaged in poultry-farming. Future studies on economic and social geography (e.g., addressing the modes of inter-provincial poultry transport) in Indonesia might help to further elucidate the effect on the viral dispersal by human, agricultural and industrial activities. In this study, we opted for a lower geographical resolution (i.e., four widely ranged geographical states instead of distinct geographical coordinate for each viral isolate) in our phylogeographic analyses because of the varying precision of the geographical data we have. Therefore, more complex hypotheses of viral origin and migration trajectory cannot be investigated here, but can be explored when more high-quality geographical data of Indonesian H5N1 viral samples is available.

The BSP analyses (Figure 3E–3H) indicate that the reassortant viruses follow a logistic-like growth curve, which is typical for virus invasion and maintenance, especially in a structured population [24],[25]. In contrast, the group 2 viruses followed a more continuous and relatively slow growth in diversity. There was insufficient data in our samples to definitively discriminate between alternative population growth models and provide narrow confidence intervals for parameter estimates, but our results are suggestive and future sequencing will add to the needed statistical power.

What factors have contributed to the apparent difference in the growth of genetic diversity? Rates of molecular evolution between the two groups were similar (Figure S6) and therefore are not likely to be the cause. Since our analyses could not resolve the temporal dynamics of population subdivision by geography, we cannot directly investigate how viral genetic variation is affected by the population structure. We expect future development of analysis methods will help to shed more light on the interaction between viral migration and genetic diversity.

Analysis of clinical records (Table 1) found that the mean duration from onset to death in those fatal human cases caused by Indonesian reassortant H5N1 viruses is 9.1 days (standard deviation [SD] = 3.9; n = 23) and those caused by other Indonesian H5N1 viruses is 7.7 days (SD = 2.7; n = 10), and their means are not significantly different (student *t*-test, *p*>0.25, two-tails). Therefore, based on the clinical records, the reassortant viruses did not kill human faster than other Indonesian H5N1 viruses did. However, we would recommend more experimental studies addressing the virulence, pathogenicity and immunogenicity of the reassortant viruses and the parent strains to verify this claim in the future.

Mechanisms of viral transmissions are sometimes correlated with genetic diversity dynamics. For example, hepatitis C viruses transmitted by drug injection or blood products have a faster rate of spread than endemic strains circulating in Asia and Africa [32]. It has also been suggested that mosquito susceptibility may affect the growth of dengue viruses [33]. Therefore, it is possible that a change of host species could generate the difference in the viral dynamics we observe. In our study, the majority of the reassortant viruses (24/25) were isolated from humans, whereas only a minority of the group 2 viruses were isolated from humans (10/57 and 10/41 in the *HA* and *NA* datasets, respectively).

It has been previously shown that the receptor binding specificity of hemagglutinin [34] and mutations in the viral polymerase (e.g., lysine at residue 627 of PB2) [35]–[37] can determine viral transmissibility and replication in different host species. None of the aforementioned *HA* mutations which confer recognition to human-type host cell receptors [34],[38] were found in the Indonesian reassortant viruses; however, our detection of positively selected sites in the *PB2* gene of the reassortant viruses could potentially reflect adaptation to mammalian hosts, and requires further investigation. In particular, amino acid changes on two positively selected sites (threonine to methionine at reside 76, glutamic acid to glycine and alanine at reside 677) were found on the internal branches of the reassortant lineage, corresponding to molecular changes during sustainable transmissions. However, some of these positively selected changes may also result from the compensatory evolution as the mix of genome segments from different strains might alter their epistatic physiochemistry [39]. Although most of the human isolates in our datasets were epidemiologically unlinked, such linkage is theoretically possible if many asymptotic or mildly manifested human infections are not reported. Recently, some evidence of subclinical or asymptotic H5N1 infection in humans has been put forward [40],[41]; however, the ability of the viruses to transmit from these infected individuals to other susceptible individuals remains unknown.

The possible role of other animal host species in the transmissions of reassortant viruses in Indonesia should not be neglected. In particular, one of the reassortant viruses was isolated from a dead cat in Jakarta, where H5N1 outbreaks in poultry and sporadic human infections have been reported [42]. Moreover, unusual high mortality of cats in the vicinity of H5N1 HPAI outbreaks has been reported [43]. An unofficial report also detected H5N1 HPAI sero-positivity in around 20% of 500 blood samples taken from stray cats near poultry markets in Java and Sumatera [44]. In addition to small cats in Germany [45], Iran [46], and Indonesia [42], dogs and zoo tigers were also found infected with H5N1 HPAI viruses in Thailand [47],[48]. Furthermore, previous experimental studies have demonstrated that cats can be infected with H5N1 HPAI virus [49],[50], and that cat-to-cat transmission is possible [49],[51]. Could cats, or other non-avian species, have played a role in spreading the reassortant viruses in Java? Similarly, could cats act as amplifying hosts facilitating viral expansion and cross-species transmission, as civets did in the SARS outbreaks [52]? Future experimental studies on these reassortant viruses, that assess viral transmissibility between species, together with epidemiological studies, such as viral monitoring within Indonesian animal populations using serological tests and PCR detection, would give more clues to these questions.

H5N1 HPAI viruses have been endemic and evolved into different genetic lineages that have spread across Indonesia. Areas where more than one lineage of virus is co-circulating, such as Jakarta, are most likely to generate novel viruses by inter-lineage reassortment. These reassortant viruses have distinctive evolutionary and transmission dynamics, as shown in this study. We suggest that more intensive and timely field surveillance and analysis of influenza viruses, including H5N1 HPAI and human H3N2, H1N1, and H1N2 epidemic strains, should be employed, so that bio-security can be undertaken promptly and appropriate strains can be selected for vaccine production whenever a novel reassortant strain emerges. The reassortant viruses reported in this study should be also added to the watch list for the future epidemiological surveillance.

## Materials and Methods

### Sequence data collection and alignment

H5N1 influenza viruses isolated from avian and mammalian hosts in Indonesia during 2003–2007 were studied. Their genomic sequences (n = 807) were extracted from the Influenza Virus Resource [53] and the Influenza Sequence Database [54] in September 2007, and aligned using MUSCLE version 3.6 [55]. Columns with gaps were removed from the alignments, and sequences from the same virus strain (duplicated submission in the two databases) were filtered such that one copy was retained. Eight genome segment alignment datasets (*PB2*, *PB1*, *PA*, *HA*, *NP*, *NA*, *MP*, and *NS*), as well as four coding sequences (*M1*, *M2*, *NS1*, and *NS2*), were generated. Full details of our datasets can be found in Table S6 and S7.

### Phylogenetic analyses

Phylogenetic trees of 12 alignment datasets were reconstructed using the ML approach implemented in PhyML 3.412 [56]. The robustness of the ML tree topology was assessed by comparing the ML topology with the topologies sampled in the BMCMC analysis performed in MrBayes version 3.1.2 [57], and with bootstrapping analyses of 1,000 pseudo-replicate datasets. ML and NJ trees were estimated from the bootstrap datasets using PhyML [56] and PAUP* version 4beta10 [58], respectively. A general-time-reversal (GTR) substitution model with gamma distributed rate heterogeneity of 4 rate categories (Γ_4_) and a proportion of invariable sites were used in all tree reconstruction methods. Phylogenies were rooted with the H5N1 HPAI strain A/Ck/HK/YU324/2003, which is genetically close to the newly reported Hunan strains [17], and shares comparable genetic proximity to Indonesian clade.

### Recombination and reassortment detection

Homologous recombination within each gene segment among Indonesian H5N1 isolates was extensively searched using Recombination Detection Program version 2 (RDP2) [59], and the datasets are found to be free of homologous recombination. Putative reassortant viruses were preliminarily identified by their topological incongruity across the phylogenies of different gene segments. This was further investigated using a smaller set of Indonesian H5N1 virus isolates with full genome sequences, which included sequences of early viruses (n = 2), group 1–3 lineages (n = 12) and putative reassortant viruses (n = 10). The eight gene segment alignments were manually concatenated in the order of their length to generate a single alignment of complete genome sequences, and was further analyzed using 1) similarity plots and 2) bootscan analyses [60] implemented in SIMPLOT version 3.5.1 [61], and 3) GARD [62] available via the Datamonkey website [63]. The hypothesis of reassortment was supported if the recombinant breakpoints were detected near the junctions where the genome segments were manually concatenated.

### Phylogeography and migration analyses

The geographic locations of virus isolation were either obtained from the sequence databases, or obtained through personal communication with Catherine Smith (from Disease Control and Prevention, Atlanta, USA), or inferred from their strain names (Tables 1 and S6). The locations of isolates were indicated on the map of main island of Java in Indonesia (Figure 4). Due to the limit of our geographical data, the localities of the isolates shown in the map (Figure 4) should be regarded as arbitrary within the province which is the highest precision level shared by all viral samples. Each of the reassortant viruses was assigned with a state of either Greater Jakarta (surroundings included) or West Java depending on its place of origin (Table 1). The migratory history of the reassortant viruses (n = 25) between these geographical states were inferred based on the refined ML phylogeny of *HA* and *NA* (Figure S4) independently using two parsimony optimization methods, called ACCTRAN (accelerated transformation) and DELTRAN (delayed transformation) implemented in PAUP* software. The geographical states of all ancestral nodes in the tree were estimated to achieve minimum state changes in overall, and therefore the number of state changes and state of the MRCA of the reassortant was obtained. Polytomies were randomly resolved 1,000 times, and state changes were estimated separately for each resolution. The mean number of state changes was then calculated. To test against the null hypothesis of completely unrestricted migration between geographical states (panmixis), the mean number of observed state changes was compared with the frequency distribution of the mean number of expected state changes under the null hypotheses. The null distribution and critical values were generated by randomly shuffling the states of isolates 5,000 times (the Slatkin-Maddison test [22],[24],[64]). The migratory history of group 2 viruses was also studied using the *HA* gene in a similar manner, while each group 2 virus was assigned to either of four widely ranged geographical states: Greater Jakarta and surroundings, the rest of Java, Sumatra, and Sulawesi Selatan, including Papua. This assignment scheme is comparable to that of reassortant viruses, as West Java is part of Java.

### Estimating the rate of evolution and genetic diversity dynamics

Parameters of codon-partitioned substitution rates, demographic functions, tMRCA and tree topologies were co-estimated from *HA* and *NA* gene datasets of reassortant and group 2 viruses separately in a BMCMC framework [18] using BEAST version 1.4.6 [65]. Substitution model HKY+Γ_4_ with invariable site portion was used. Isolation dates were used to calibrate the molecular clock. Three clock models including strict clock, UCEN and UCLN relaxed clocks [21] were attempted independently, and the best-fit clock model was selected by comparing the BF calculated from their posterior distributions [20]. The Bayesian skyline plot [23] was used to estimate population dynamics, in terms of relative genetic diversity. Less complex parametric demographic models (constant size, exponential growth and logistic growth) were applied independently, and the best-fit models selected by BF tests were used to quantitatively estimate the growth rate and other demographic parameters. The BMCMC analyses contained 2×10^8^ states, with sampling every 1,000 states, and the first 10% of each chain was discarded as burn-in. Convergences and effective sample sizes of the estimates were checked using Tracer v1.4 [66].

### Detecting positively selected sites and lineages

Positively selected sites were detected using random effect likelihood (REL) and fixed effect likelihood (FEL) methods [26] via the Datamonkey website [63]. Bayes factors larger than 50 and *p*-values smaller than 0.1 were used as thresholds for strong evidence of selection in REL and FEL, respectively. To test lineage-specific positive selection, the two-ratio branch model was used, which pre-specifies a single rate of synonymous substitution (dS) for the whole phylogeny and two rates of non-synonymous substitution (dN_1_ and dN_2_). The dN_1_ was specified for the pre-emergence lineage (indicated as the ancestral branch connecting the group 3 MRCA; see Figure 1B) for the group 3 viruses (including the reassortant viruses for *M1*, *M2*, and *PB1* genes). The dN_2_ was specified for other lineages across the phylogenies. The ML estimates of these rate parameters were performed in HYPHY version 0.99 [67]. The resulting likelihood score of the two-ratio model was then compared with that of the one-ratio model, which assumes the same dN and dS across the phylogeny, using the likelihood ratio test (LRT, with degree of freedom = 1). The substitution model MG94XGTR+Γ_4_ was used.

### Identifying mutations fixed along the lineage

The ancestral nucleotide sequences of all internal nodes were reconstructed using joint ML method [68] implemented in HYPHY. Amino acid changes along the pre-emergence lineage were determined, and were then mapped onto the three-dimensional structure of the N-terminal domain of M1 matrix protein molecule [30] available (PDB-ID: 1EA3) in RCSB Protein Data Bank.

## Supporting Information

Figure S1 Phylogenies of *PB2* and *PB1* genes of Indonesian H5N1 HPAI viruses. ML phylogenies reconstructed from (A) *PB2* gene and (B) *PB1* gene. Topological supports (>90) summarized from 1,000 ML bootstrap replications are shown. For major lineages, NJ bootstrap support (1,000 replications) and posterior probability from BMCMC analyses (5,000 tree samples) are also shown inside parentheses (ML/NJ/BMCMC). Putative human and cat reassortant viruses are in red. Putative avian reassortant viruses are in blue. Arrows indicate the roots. Distance unit is substitutions/site.
(2.81 MB EPS)

Figure S2 Phylogenies of *PA*, *NP*, and *NA* genes of Indonesian H5N1 HPAI viruses. ML phylogenies reconstructed from (A) *PA* gene, (B) *NP* gene, and (C) *NA* gene. Topological supports (>90) summarized from 1,000 ML bootstrap replications are shown. For major lineages, NJ bootstrap support (1,000 replications) and posterior probability from BMCMC analyses (5,000 tree samples) are also shown inside parentheses (ML/NJ/BMCMC). Putative human and cat reassortant viruses are in red. Putative avian reassortant viruses are in blue. Arrows indicate the roots. Distance unit is substitutions/site.
(3.06 MB EPS)

Figure S3 Phylogenies of MP and NS segments, *NS1* and *NS2* genes of Indonesian H5N1 HPAI viruses. ML phylogenies reconstructed from (A) MP segment, (B) NS segment, (C) *NS1* gene, and (D) *NS2* gene. Topological supports (>90) summarized from 1,000 ML bootstrap replications are shown. For major lineages, NJ bootstrap support (1,000 replications) and posterior probability from BMCMC analyses (5,000 tree samples) are also shown inside parentheses (ML/NJ/BMCMC). Putative human and cat reassortant viruses are in red. Arrows indicate the roots. Distance unit is substitutions/site.
(3.19 MB EPS)

Figure S4 Refined phylogenies of reassortant viruses inferred from their *HA* and *NA* genes. ML phylogenies reconstructed from (A) *HA* gene and (B) *NA* gene. Topological supports summarized from 1,000 ML bootstrap replications are shown. For major lineages, NJ bootstrap support (1,000 replications) and posterior probability from BMCMC analyses (5,000 tree samples) are also shown inside parentheses (ML/NJ/BMCMC). Subgroups R1, R2, and R3 reassortant viruses are in red, blue, and green, respectively. Arrows indicate the rooting using strain IDN/5/05 as the outgroup. Distance unit is substitutions/site.
(1.18 MB EPS)

Figure S5 Mean number of observed geographical state changes and the frequency distribution of mean number of expected geographical state changes under null hypothesis of panmixis. (A–D) show the frequency distribution of reassortant viruses from the HA ([A–B]) and NA ([C–D]) datasets using delayed transformation (DEL; [A,C]) and accelerated transformation (ACC; [B,D]) methods. (E) and (F) show the frequency distribution of group 2 viruses from the HA dataset using DEL and ACC methods, respectively. Arrowheads indicate the mean number of observed geographical state changes (GSCs) and the corresponding *p*-value tested against the null distribution.
(1.24 MB EPS)

Figure S6 Substitution rates of *HA* and *NA* genes from reassortant and group 2 parental strains. 95% higher probability densities (HPDs) are indicated by the error bars. 1st, 2nd, 3rd, and C denote the rate for the 1st codon position, 2nd codon position, 3rd codon position, and whole sequence (non-partitioned), respectively. Substitution rate units for codon partitioned and non-partitioned sequences are substitution/codon/year and substitution/site/year, respectively.
(0.88 MB EPS)

Figure S7 Molecular structure of the dimer formed by two N-terminal domains of the M1 matrix protein. There are 8 snapshots of the M1 dimer structure (PDB-ID: 1EA3) in which each is rotated 45 degrees counter-clockwise (on x-plane) from its left snapshot. Blue and red indicate the positively and negatively charged surfaces, respectively. The residues in which the substitutions occurred along the pre-emergence lineage are highlighted with green (residues 37, 95, and 137 are indicated by white, orange, and yellow arrows, respectively). The structure is visualized by DeepView software.
(3.54 MB TIF)

Figure S8 Growth curves of genetic diversity estimated by simple parametric models are superimposed on Bayesian skyline plots.
(2.82 MB EPS)

Dataset S1 Computer files of phylogenetic trees of Indonesian H5N1 influenza viruses. The zip file includes dendrogram (in NEWICK format) of ML phylogenies reconstructed from *PB2*, *PB1*, *PA*, *HA*, *NP*, *NA*, *MP*, *M1*, *M2*, *NS*, *NS1*, and *NS2* gene datasets (described in main text). Topological supports (percentages) shown in the internal nodes were summarized from 1,000 ML bootstrap replications. NJ bootstrap replications and BMCMC sampled trees are available upon request.
(0.02 MB ZIP)

Table S1 Parameter estimates of best-fit parametric demographic models in *HA* and *NA* gene datasets of reassortant and its parental strain. 95% highest probability densities (HPDs) of the estimates are shown in the parentheses.
(0.04 MB DOC)

Table S2 Proportion of geographical state of the MRCAs of reassortant viruses inferred from 1,000 polytomy-resolved trees by parsimony methods. Ambiguous states estimated are ignored. Two parsimony optimizations, including delayed transformation (DEL) and accelerated transformation (ACC), were used.
(0.04 MB DOC)

Table S3 Difference between mean observed and expected number of geographical state changes in the parental strain viruses (group 2).
(0.05 MB DOC)

Table S4 Bayes factor testing of different molecular clock and demographic models in BMCMC analyses. Underlined are the selected best-fit models that could not be rejected by the alternative models.
(0.05 MB DOC)

Table S5 Estimations of dN/dS using 1-ratio and 2-ratio lineage-specific selection models. These estimations were performed in HYPHY software. Gene datasets other than *PB1*, *HA*, *NA*, *M1*, and *M2* were not analyzed because group 3 is represented by the single virus IDN/6/05.
(0.03 MB DOC)

Table S6 Information and phylogenetic groupings of sequences used in this study. 1, 2, 3, and X denotes groups 1, 2, 3, and unclassified (early viruses; see main text for explanation). Empty entries indicate the unavailability (e.g., no sequence found, too short, too many ambiguous codes, and too many gaps) of the sequence.
(0.35 MB DOC)

Table S7 Accession numbers of the sequences used in this study.
(0.93 MB DOC)
