# Supplementary figures and images for: Evolutionary and Transmission Dynamics of Reassortant H5N1 Influenza Virus in Indonesia
Source: PLoS Pathog. 2008 Aug 22;4(8):e1000130. doi: 10.1371/journal.ppat.1000130 (PMC2515348; doi:10.1371/journal.ppat.1000130)

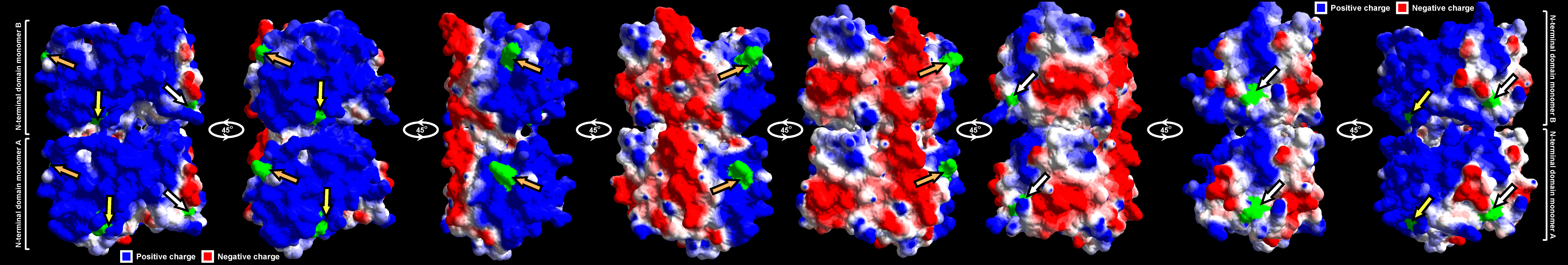

Supplement: Figure S7 — Molecular structure of the dimer formed by two N-terminal domains of the M1 matrix protein. There are 8 snapshots of the M1 dimer structure (PDB-ID: 1EA3) in which each is rotated 45 degrees counter-clockwise (on x-plane) from its left snapshot. Blue and red indicate the positively and negatively charged surfaces, respectively. The residues in which the substitutions occurred along the pre-emergence lineage are highlighted with green (residues 37, 95, and 137 are indicated by white, orange, and yellow arrows, respectively). The structure is visualized by DeepView software. (3.54 MB TIF) [file ppat.1000130.s007.tif]
