# Supplementary material for: Evolutionary and Transmission Dynamics of Reassortant H5N1 Influenza Virus in Indonesia
Source: PLoS Pathog. 2008 Aug 22;4(8):e1000130. doi: 10.1371/journal.ppat.1000130 (PMC2515348; doi:10.1371/journal.ppat.1000130)
Supplement: Table S1 — Parameter estimates of best-fit parametric demographic models in HA and NA gene datasets of reassortant and its parental strain. 95% highest probability densities (HPDs) of the estimates are shown in the parentheses. (0.04 MB DOC) [file ppat.1000130.s010.doc]

**Table S1. Parameter estimates of best-fit parametric demographic models in HA and NA gene datasets of reassortant and its parental strain. 95% highest probability densities (HPDs) of the estimates are shown in the parentheses.**

| **Virus group** | Parental strain (Group 2) | | | | | |  | Reassortant strain | | | | | |
| --- | --- | --- | --- | --- | --- | --- | --- | --- | --- | --- | --- | --- | --- |
| **Gene** | HA | | | NA | | |  | HA | | | NA | | |
| **Model**^a^ | CONST | EXPO | LOG | CONST | EXPO | LOG |  | CONST | EXPO | LOG | CONST | EXPO | LOG |
| **Posterior lnL** | -4146.971 | -4143.03 | -4141.815 | -2930.281 | -2927.61 | -2928.835 |  | -2938.848 | -2936.662 | -2931.988 | -2248.844 | -2241.904 | -2239.774 |
| **Pop. size (N_e_τ)** | 2.212  (1.386 - 3.401) | 6.065  (1.349 - 16.71) | 2.701  (1.429 - 5.175) | 2.269  (1.174 - 3.921) | 9.555  (1.178 - 44.518) | 3.240  (0.897 - 2435.782) |  | 0.785  (0.362 - 1.404) | 2.648  (0.252 - 13.578) | 1.085  (0.408 - 2.54) | 0.995  (0.396 - 1.902) | 7.861  (0.246 - 100.383) | 1.616  (0.36 - 8.549) |
| **Growth rate (year^-1^)** |  | 0.844  (0.087 - 1.683) | 7.057  (0.039 - 22.467) |  | 1.071  (0.072 - 2.257) | 7.377  (0.001 - 23.156) |  |  | 1.613  (-0.344 - 3.782) | 11.682  (2.091 - 24.868) |  | 2.820  (0.050 - 5.847) | 7.995  (0.017 - 22.662) |
| **Doubling time (year)** |  | 0.811  (0.270 - 3.098) | 0.098  (0.028 - 0.610) |  | 0.640  (0.200 - 2.481) | 0.094  (0.028 - 2.679) |  |  | 0.399  (-2.431 - 3.857) | 0.059  (0.028 - 0.315) |  | 0.244  (0.058 - 0.928) | 0.087  (0.028 - 0.564) |
| **t50**^b^ **(year)** |  |  | 2.054  (0.668 - 4.158) |  |  | 2.281  (0.000 - 4.487) |  |  |  | 1.14  (0.543 - 1.986) |  |  | 1.008  (0.002 - 2.289) |

^a^ CONST denotes constant population model; EXPO denotes exponential growth model; LOG denotes logistic growth model.

^b^ t50 denotes the time before the date of most recent sample that the population size was 50% of maximum in logistic growth model.
