# Supplementary material for: Evolutionary and Transmission Dynamics of Reassortant H5N1 Influenza Virus in Indonesia
Source: PLoS Pathog. 2008 Aug 22;4(8):e1000130. doi: 10.1371/journal.ppat.1000130 (PMC2515348; doi:10.1371/journal.ppat.1000130)
Supplement: Table S2 — Proportion of geographical state of the MRCAs of reassortant viruses inferred from 1,000 polytomy-resolved trees by parsimony methods. Ambiguous states estimated are ignored. Two parsimony optimizations, including delayed transformation (DEL) and accelerated transformation (ACC), were used. (0.04 MB DOC) [file ppat.1000130.s011.doc]

**Table S2. Proportion of geographical state of the MRCAs of reassortant viruses inferred from 1000 polytomy-resolved trees by parsimony methods.** Ambiguous states estimated are ignored. Two parsimony optimizations, including delayed transformation (DEL) and accelerated transformation (ACC) were used.

|  |  | **Delayed transformation (DEL)** | |  | **Accelerated transformation (ACC)** | |
| --- | --- | --- | --- | --- | --- | --- |
| **Gene** | **MRCA** | **G. Jakarta & surroundings** | **West Java** |  | **G. Jakarta & surroundings** | **West Java** |
| HA | All-MRCA | 0.971 | 0.029 |  | 1 | 0 |
|  | R1-MRCA | 0.972 | 0.028 |  | 1 | 0 |
|  | R2-MRCA | 0.923 | 0.077 |  | 0.919 | 0.081 |
|  | R3-MRCA | 0.933 | 0.067 |  | 0.795 | 0.205 |
| NA | All-MRCA | 0.965 | 0.035 |  | 0.974 | 0.026 |
|  | R1-MRCA | 0.966 | 0.034 |  | 0.966 | 0.034 |
|  | R2-MRCA | 0.965 | 0.035 |  | 0.974 | 0.026 |
|  | R3-MRCA | 0.975 | 0.025 |  | 0.959 | 0.041 |
