# Supplementary material for: Evolutionary and Transmission Dynamics of Reassortant H5N1 Influenza Virus in Indonesia
Source: PLoS Pathog. 2008 Aug 22;4(8):e1000130. doi: 10.1371/journal.ppat.1000130 (PMC2515348; doi:10.1371/journal.ppat.1000130)
Supplement: Table S3 — Difference between mean observed and expected number of geographical state changes in the parental strain viruses (group 2). (0.05 MB DOC) [file ppat.1000130.s012.doc]

**Table S3. Difference between mean observed and expected number of geographical state changes in the parental strain viruses (group 2).**

| **Type of data** |  | **Place of destination^a^** | | | | | | | | |
| --- | --- | --- | --- | --- | --- | --- | --- | --- | --- | --- |
|  |  | **Delayed transformation (DEL)** | | | |  | **Accelerated transformation (ACC)** | | | |
|  | **Place of origin^a^** | **JAKS** | **JAVA** | **SMRA** | **SWSP** |  | **JAKS** | **JAVA** | **SMRA** | **SWSP** |
| **Observed (O) ^d^** | **JAKS** |  | 2.106 | 1.978 | 0.484 |  |  | 1.282 | 2.815 | 0 |
|  | **JAVA** | 5.378 |  | 3.883 | 0.516 |  | 5.548 |  | 2.846 | 0.351 |
|  | **SMRA** | 0.224 | 0.915 |  | 0 |  | 0.339 | 1.491 |  | 0.649 |
|  | **SWSP** | 0 | 0 | 0 |  |  |  | 0.163 | 0 |  |
| **Expected (E) ^e^** | **JAKS** |  | 0.384 | 0.395 | 0.168 |  |  | 0.923 | 1.347 | 0.624 |
|  | **JAVA** | 5.984 |  | 9.522 | 3.831 |  | 5.159 |  | 7.366 | 2.58 |
|  | **SMRA** | 0.894 | 1.69 |  | 0.696 |  | 1.173 | 2.606 |  | 1.253 |
|  | **SWSP** | 0.049 | 0.05 | 0.073 |  |  | 0.112 | 0.402 | 0.158 |  |
| **O - E ^f^** | **JAKS** |  | 1.722 **^b^** | 1.583 **^b^** | 0.316 |  |  | 0.359 | 1.468 **^c^** | -0.624 |
|  | **JAVA** | -0.606 |  | -5.639 **^b^** | -3.315 **^b^** |  | 0.389 |  | -4.52 **^b^** | -2.229 **^b^** |
|  | **SMRA** | -0.67 | -0.775 |  | -0.696 |  | -0.834 | -1.12 |  | -0.604 |
|  | **SWSP** | -0.049 | -0.05 | -0.073 |  |  | -0.112 | -0.24 | -0.158 |  |

**^a^** JAKS denotes Greater Jakarta and surroundings; JAVA denotes the rest of Java; SMRA denotes Sumatera; SWSP denotes Sulawesi Selatan and Papua.

**^b^** p < 0.05 when the observed value of this migratory direction is compared to the distribution of expected value of this migratory direction.

**^c^** p < 0.1 when the observed value of this migratory direction is compared to the distribution of expected value of this migratory direction.

**^d^** Estimated from HA gene dataset.

**^e^** Estimated from 5000 datasets with taxa randomly shuffled.

**^f^** Positive difference suggests migration; Negative difference suggests relative isolation.
