# Supplementary material for: Evolutionary and Transmission Dynamics of Reassortant H5N1 Influenza Virus in Indonesia
Source: PLoS Pathog. 2008 Aug 22;4(8):e1000130. doi: 10.1371/journal.ppat.1000130 (PMC2515348; doi:10.1371/journal.ppat.1000130)
Supplement: Table S4 — Bayes factor testing of different molecular clock and demographic models in BMCMC analyses. Underlined are the selected best-fit models that could not be rejected by the alternative models. (0.05 MB DOC) [file ppat.1000130.s013.doc]

**Table S4. Bayes factor testing of different molecular clock and demographic models in BMCMC analyses. Underlined are the selected best-fit models which could not be rejected by the alternative models.**

|  |  | **Test molecular clocks** | | | | | **Test parametric demographic models** | | | |
| --- | --- | --- | --- | --- | --- | --- | --- | --- | --- | --- |
| **Virus group** | **Gene** | **Clock model ^a^** | **Marginal lnL^b^** | **Comparison** | **lnBF^c^** |  | **Demo. model ^d^** | **Marginal lnL^b^** | **Comparison** | **lnBF^c^** |
| Reassortant | HA | CLOCK | -2925.9077 | CLOCK VS UCED | 4.1183 |  | CONS | -2921.8203 | CONS VS EXPO | 0.1410 |
|  |  | UCED **^e^** | -2921.7894 | CLOCK VS UCLD | 1.508 |  | EXPO | -2921.9613 | CONS VS LOG | 0.2949 |
|  |  | UCLD | -2924.3997 | UCED VS UCLD | 2.6103 |  | LOG | -2921.5254 | EXPO VS LOG | 0.4359 |
|  | NA | CLOCK | -2225.2452 | CLOCK VS UCED | 0.2944 |  | CONS | -2225.7350 | CONS VS EXPO | 0.0707 |
|  |  | UCED | -2224.9508 | CLOCK VS UCLD | 0.2225 |  | EXPO | -2225.8057 | CONS VS LOG | 0.2918 |
|  |  | UCLD | -2225.0227 | UCED VS UCLD | 0.0719 |  | LOG | -2225.4432 | EXPO VS LOG | 0.3625 |
| Parent (group 2) | HA | CLOCK | -4085.0921 | CLOCK VS UCED | 33.2195 |  | CONS | -4051.9602 | CONS VS EXPO | 0.0751 |
|  |  | UCED | -4051.8726 | CLOCK VS UCLD | 27.4925 |  | EXPO | -4051.8851 | CONS VS LOG | 0.0553 |
|  |  | UCLD | -4057.5996 | UCED VS UCLD | 5.727 |  | LOG | -4051.9049 | EXPO VS LOG | 0.0198 |
|  | NA | CLOCK | -2878.9753 | CLOCK VS UCED | 15.0335 |  | CONS | -2863.5714 | CONS VS EXPO | 0.1912 |
|  |  | UCED | -2863.9418 | CLOCK VS UCLD | 10.1535 |  | EXPO | -2863.7626 | CONS VS LOG | 0.5957 |
|  |  | UCLD | -2868.8218 | UCED VS UCLD | 4.88 |  | LOG | -2862.9757 | EXPO VS LOG | 0.7869 |

^a^ Molecular clock models: CLOCK denotes strict clock model; UCED denotes uncorrelated exponential distributed relaxed clock model; UCLD denotes uncorrelated lognormal distributed relaxed clock model.

^b^ An importance sampling estimator of the marginal likelihood was employed according to Suchard et al. (2003).

^c^ lnBF denotes natural log Bayes factor which is the difference of natural log marginal likelihoods between models in test; lnBF > 2.99 is considered to be significantly different.

^d^ Parametric demographic models: CONS denotes constant growth model; EXPO denotes exponential growth model; LOG denotes logistic growth model. All parametric demographic models fit almost equally well (lnBF < 1) in all viral datasets.

^e^ Although UCED did not significantly outperform UCLD (slightly less than the threshold, 2.99), it significantly outperformed CLOCK whereas UCLD did not.

*References:* Suchard MA, Kitchen CM, Sinsheimer JS, Weiss RE (2003) Hierarchical phylogenetic models for analyzing multipartite sequence data. Syst Biol 52: 649-664.
