# Supplementary material for: Evolutionary and Transmission Dynamics of Reassortant H5N1 Influenza Virus in Indonesia
Source: PLoS Pathog. 2008 Aug 22;4(8):e1000130. doi: 10.1371/journal.ppat.1000130 (PMC2515348; doi:10.1371/journal.ppat.1000130)
Supplement: Table S5 — Estimations of dN/dS using 1-ratio and 2-ratio lineage-specific selection models. These estimations were performed in HYPHY software. Gene datasets other than PB1, HA, NA, M1, and M2 were not analyzed because group 3 is represented by the single virus IDN/6/05. (0.03 MB DOC) [file ppat.1000130.s014.doc]

**Table S5. Estimations of dN/dS using one-ratio and two-ratios lineage-specific selection models.** These estimations were performed in HYPHY software. Gene datasets other than PB1, HA, NA, M1 and M2 were not analyzed because group 3 is represented by a single virus IDN/6/05 there.

| **Gene** | **One-ratio model lnL** | **Two-ratios model lnL** | **p-value ^a^** | **dN_1_/dS ^b^** | **dN_2_/dS ^c^** |
| --- | --- | --- | --- | --- | --- |
| PB1 | -3530.6471 | -3529.8274 | 0.200 | 0.090 |  |
| HA* | -7086.2513 | -7085.0778 | 0.126 | 0.170 |  |
| NA | -4135.1457 | -4135.1427 | 0.938 | 0.222 |  |
| M1 | -1667.1089 | -1662.5957 | 0.003 | 0.077 | 1.514 (0.447 - 3.814) |
| M2 | -622.907 | -621.9453 | 0.166 | 1.023 |  |

**^a^** When p-value < 0.05, two-ratios model was considered significantly outperforming the one-ratio model, and was used for dN/dS estimation..

**^b^** dN**_1_**/dS denotes the dN across the whole phylogeny divided by phylogeny-common dS when one-ratio model was selected and used; dN**_1_**/dS denotes the dN across all lineages other than the pre-emergence lineage of group 3 viruses, divided by phylogeny-common dS if two-ratios model was selected and used.

**^c^** dN**_2_**/dS denotes the dN of pre-emergence lineage of group 3 viruses divided by phylogeny-common dS if two-ratios model was selected and used. Confident interval is shown inside parenthesis; dN**_2_**/dS is not present in one-ratio model.
