# Supplementary material for: Evolutionary and Transmission Dynamics of Reassortant H5N1 Influenza Virus in Indonesia
Source: PLoS Pathog. 2008 Aug 22;4(8):e1000130. doi: 10.1371/journal.ppat.1000130 (PMC2515348; doi:10.1371/journal.ppat.1000130)
Supplement: Table S6 — Information and phylogenetic groupings of sequences used in this study. 1, 2, 3, and X denotes groups 1, 2, 3, and unclassified (early viruses; see main text for explanation). Empty entries indicate the unavailability (e.g., no sequence found, too short, too many ambiguous codes, and too many gaps) of the sequence. (0.35 MB DOC) [file ppat.1000130.s015.doc]

**Table S6. Information and phylogenetic groupings of sequences used in this study.** Symbol 1, 2, 3 and X denotes group 1, 2, 3 and unclassified (early viruses, see main text for explanation). Empty entry indicates the unavailability (e.g. no sequence found, too short, too many ambiguous codes, and too many gaps) of the sequence.

| **Strain name** | **NS** | **MP** | **NA** | **NP** | **HA** | **PA** | **PB1** | **PB2** |
| --- | --- | --- | --- | --- | --- | --- | --- | --- |
| Ck/Agam/BBPVI/05(Ck/Agam/BBPV1/05) |  |  | 1 |  | 1 |  |  |  |
| Ck/Bandar Lampung/BBPVIII/06  (Ck/Bandar Lampung/BPPV111/06) |  |  | 2 |  | 2 |  |  |  |
| Ck/Bangli Bali/BBPV6 1/04 | X | X | X | X | X | X | X | X |
| Ck/Bangli Bali/BPPV6 2/04 | X | X | X | X | X | X | X | X |
| Ck/Bantul/BBVet I/05 | X | X | X | X | X |  | X | X |
| Ck/Dairi/BPPVI/05 | 1 | 1 | 1 | 1 | 1 | 1 | 1 | 1 |
| Ck/Deli Serdang/BPPVI/05  (Ck/Deli Serdang/BPPV1/05)  (Ck/Deli Derdang/BBPVI/05)  (Ck/Deli Derdang/BBPV1/05) | 1 | 1 | 1 | 1 | 1 | 1 | 1 | 1 |
| Ck/Duma/BBPV II/05  (Ck/Duma/BPPV11/05) |  |  | 1 |  | 1 |  |  |  |
| Ck/Gunung Kidal/BBVW/05 | 2 | 2 | 2 | 2 | 2 | 2 | 2 | 2 |
| Ck/Gunung Kidul/BBVW/06 |  |  |  |  | 3 |  |  |  |
| Ck/IDN/11/03 |  | X | X |  | X |  |  |  |
| Ck/IDN/2A/03 | X | X | X | X | X | X | X | X |
| Ck/IDN/4/04 | X | X | X | X | X | X | X | X |
| Ck/IDN/5/04 | X | X | X | X | X | X | X | X |
| Ck/IDN/7/03 |  | X | X |  | X |  |  |  |
| Ck/IDN/Agam1631 3/06 |  |  | 2 |  | 2 |  |  |  |
| Ck/IDN/Bandung1631 49/06 |  |  | 2 |  | 2 |  |  |  |
| Ck/IDN/Bangka Seletan1631 20/06 |  |  | 2 |  | 2 |  |  |  |
| Ck/IDN/Bangka Seletan1631 21/06 |  |  | 2 |  | 2 |  |  |  |
| Ck/IDN/Belitung Timor1631 18/06 |  |  | 2 |  | 2 |  |  |  |
| Ck/IDN/BL/03 | X | X | X | X | X | X | X | X |
| Ck/IDN/CDC24/05 | 2 | 2 | 2 |  | 2 | 2 | 2 | 2 |
| Ck/IDN/CDC25/05 | 2 | 2 | 2 | 2 | 2 | 2 | 2 | 2 |
| Ck/IDN/Garut1631 51/06 |  |  | 2 |  | 2 |  |  |  |
| Ck/IDN/Gunung Kidul1631 33/06 |  |  | 2 |  | 2 |  |  |  |
| Ck/IDN/Kulon1631 47/06 |  |  | 3 |  | 3 |  |  |  |
| Ck/IDN/Lampung1631 23/06 |  |  | 2 |  | 2 |  |  |  |
| Ck/IDN/Magelang1631 57/07 |  |  | X |  | 3 |  |  |  |
| Ck/IDN/PA/03 | X | X | X | X | X | X | X | X |
| Ck/IDN/Padang1631 1/06 |  |  | 2 |  | 2 |  |  |  |
| Ck/IDN/Pekenbaru1631 11/06 |  |  | 2 |  | 2 |  |  |  |
| Ck/IDN/R134/03 |  |  | X |  |  |  |  |  |
| Ck/IDN/R60/05 |  |  | X |  | X |  |  |  |
| Ck/IDN/Rejang Lebong1631 22/06 |  |  | 2 |  | 2 |  |  |  |
| Ck/IDN/Semerang1631 62/07 |  |  | 3 |  | 2 |  |  |  |
| Ck/IDN/Siak1631 2/06 |  |  | 2 |  | 2 |  |  |  |
| Ck/IDN/Soppeng1631 71/07 |  |  |  |  | 3 |  |  |  |
| Ck/IDN/Wates1/05 |  |  | 2 |  | 2 |  |  |  |
| Ck/IDN/Wates126/05 |  |  | 2 |  | 2 |  |  |  |
| Ck/IDN/Wates130/05 |  |  | 2 |  | 2 |  |  |  |
| Ck/IDN/Wates77/05 |  |  | X |  | X |  |  |  |
| Ck/IDN/Wates80/05 |  |  | X |  | X |  |  |  |
| Ck/IDN/Wates83/05 |  |  | X |  | X |  |  |  |
| Ck/Jembrana/BPPV6/04 | X | X | X | X | X | X | X |  |
| Ck/Karo/BBPVII/06  (Ck/Karo/BBPV1/06) |  |  | 1 |  | 1 |  |  |  |
| Ck/Kulon Progo/BBVet XII 1/04 | X | X | X | X | X | X | X | X |
| Ck/Kulon Progo/BBVet XII 2/04 | X | X | X | X | X | X | X | X |
| Ck/Kulon Progo/BBVW/05 | X | X | X | X | X | 2 | X |  |
| Ck/Kupang 1 NTT/BPPV6/04 | X | X | X | X | X | X | X | X |
| Ck/Kupang 2 NTT/BPPV6/04 | X | X | X | X | X | X | X | X |
| Ck/Kupang 3 NTT/BPPV6/04 | X | X | X | X | X | X | X | X |
| Ck/Langkat/BBPV1 576/05  (Ck/Langkat/BBPV1/05) |  |  | 1 |  | 1 |  |  |  |
| Ck/Madiun/BBVW1420/05 |  |  |  |  | 3 |  |  |  |
| Ck/Magetan/BBVW/05 | 2 | 2 | 2 | 2 | 2 | 2 | 2 | 2 |
| Ck/Malang/BBVet IV/04 | X | X | X | X | X | X | X | X |
| Ck/Mangarai NTT/BPPV6/04 | X | X | X | X | X | X | X | X |
| Ck/Medan/BBPV1 498/05 |  |  | 1 |  |  |  |  |  |
| Ck/Medan/BBPV1 534/05 |  |  | 1 |  |  |  |  |  |
| Ck/Medan/BBPV1 571/05 |  |  |  |  | 1 |  |  |  |
| Ck/Medan/BBPV1 576/05 |  |  | 1 |  | 1 |  |  |  |
| Ck/Medan/BPPV1 498/05 |  |  |  |  | 1 |  |  |  |
| Ck/Medan/BPPV1 534/05 |  |  |  |  | 1 |  |  |  |
| Ck/Murao Jambi/BBPV II/05  (Ck/Murao Jambi/BPPV11/05) |  |  | 2 |  | 2 |  |  |  |
| Ck/Ngawi/BPPV4/04 | X | X | X | X | X | X | X | X |
| Ck/Padang/BBPVII/06  (Ck/Padang/BPPV11/06) |  |  | 1 |  | 1 |  |  |  |
| Ck/Pakun Baru/BPPV II/05  (Ck/Pakunbaru/BPPV11/05) |  |  | 2 |  | 2 |  |  |  |
| Ck/Palembang/BPPV III/05  (Ck/Palembang/BPPV111/05) |  |  | 2 |  | 2 |  |  |  |
| Ck/Pangkalpinang/BPPV3/04 | X | X | X | 1 | X | X | X |  |
| Ck/Papua/TA5/06 |  |  |  |  | 2 |  |  |  |
| Ck/Papua/TB1/06 |  |  |  |  | 2 |  |  |  |
| Ck/Papua/TB15/06 |  |  |  |  | 2 |  |  |  |
| Ck/Paulau Rampang/BPPV11/06 |  |  | 1 |  |  |  |  |  |
| Ck/Pekalongan/BPPV4/03 | X | X | X | X | X | X | X |  |
| Ck/Pidie/BBPV1/05(Ck/Pidie/BPPV1/05) |  |  | 1 |  | 1 |  |  |  |
| Ck/Pulau Rampang/BBPVII/06 |  |  |  |  | 1 |  |  |  |
| Ck/Purwakarta/BBVet IV/04 | X | X | X | X | X | X | X | X |
| Ck/Purworejo/BBVW/05 | X | X | X | X | X | X | X | X |
| Ck/Rokan Hilli/BPPV II/05  (Ck/Rokan Hilli/BPPV11/05) |  |  | 1 |  | 1 |  |  |  |
| Ck/Salam/BBPV II/05  (Ck/Salam/BBPV11/05) |  |  | 1 |  | 1 |  |  |  |
| Ck/Salatiga/BBVet I/05 | X | 1 | X | 1 | X | X | X | X |
| Ck/Sembawa/BPPV III/05 |  |  |  |  | 2 |  |  |  |
| Ck/Siak/BPPV II/05  (Ck/Siak/BPPV11/05) |  |  | 1 |  | 1 |  |  |  |
| Ck/Simalanggang/BPPVI/05 | 1 | 1 | 1 | 1 | 1 | 1 | 1 | 1 |
| Ck/Sragen/BPPV4/03 | X | X | X | X | X | X | X | X |
| Ck/Taput/BBPV1/05  (Ck/Taput/BBPV1 576/05) |  |  | 1 |  | 1 |  |  |  |
| Ck/Tarutung/BPPVI/05 | 1 | 1 | 1 | 1 | 1 | 1 | 1 | 1 |
| Ck/Tebing Tinggi/BPPVI/05 | 1 | 1 | 1 | 1 | 1 | 1 | 1 | 1 |
| Ck/Wajo/BBVM/05 | 2 | 2 | 2 | 2 | 2 | 2 | 2 | 2 |
| Ck/Way Kanan/BBPVIII/06  (Ck/Way Kanan/BPPV111/06) |  |  | 2 |  | 2 |  |  |  |
| Ck/West Java/GARUT MAY/06 |  |  |  |  | 3 |  |  |  |
| Ck/West Java/HAMD/06 |  |  |  |  | X |  |  |  |
| Ck/West Java/PWT WIJ/06 |  |  |  |  | 2 |  |  |  |
| Ck/West Java/SMI CSLK EB/06 |  |  |  |  | 2 |  |  |  |
| Ck/West Java/SMI CSLK EC/06 |  |  |  |  | 2 |  |  |  |
| Ck/West Java/SMI ENDRI1/06 |  |  |  |  | 2 |  |  |  |
| Ck/West Java/SMI ENDRI2/06 |  |  |  |  | 2 |  |  |  |
| Ck/West Java/SMI PAT/06 |  |  |  |  | 2 |  |  |  |
| Ck/West Java/TASIK1/06 |  |  |  |  | 2 |  |  |  |
| Ck/West Java/TASIK2/06 |  |  |  |  | 2 |  |  |  |
| Ck/West Java/TASIKSOB/06 |  |  |  |  | 2 |  |  |  |
| Ck/West Java/TASIKSOL/06 |  |  |  |  | 2 |  |  |  |
| Ck/Wonosobo/BPPV4/03 | X | X | X | X | X | X | X | X |
| Ck/Yogjakarta/BBVet IX/04 | X | X | X | X | X | X | X | 2 |
| Ct/IDN/CDC1/06 | 2 | 3 | 2 | 2 | 2 | 2 | 3 | 2 |
| Dk/Bufeleng/BPPV1/05  (Dk/IBufeleng/BPPV1/05) |  |  | X |  | X |  |  |  |
| Dk/IDN/MS/04 | X | X | X | X | X | X | X | X |
| Dk/Indramayu/BBPW109/06  (Dk/Indramayu/BBVW109/06) |  |  | 2 |  | 2 |  |  |  |
| Dk/Madiun/BBVW1358/05 |  |  | 1 |  | 1 |  |  |  |
| Dk/Pali/BBVW/05  (Dk/Pali/BBVW1358/05) |  |  | X |  | X |  |  |  |
| Dk/Parepare/BBVM/05 | 2 | 2 | 2 | 2 | 2 | 2 | 2 | 2 |
| Dk/Tabanan/BPPV1/05 |  |  |  |  | X |  |  |  |
| IDN/195H/05 | 2 | 2 | 2 | 2 | 2 | 2 | 2 |  |
| IDN/298H/06 | 2 | 2 | 2 | 2 | 2 | 2 | 2 |  |
| IDN/5/05 | 2 | 2 | 2 | 2 | 2 | 2 | 2 | 2 |
| IDN/6/05 | X | 3 | 3 | X | 3 | X | 3 | X |
| IDN/CDC1031/07 | 2 | 3 | 2 | 2 | 2 | 2 | 3 | 2 |
| IDN/CDC1032/07 | 2 | 3 | 2 | 2 | 2 | 2 | 3 | 2 |
| IDN/CDC1046/07 | 2 | 3 | 2 | 2 | 2 | 2 | 3 | 2 |
| IDN/CDC1047/07 | 2 | 3 | 2 | 2 | 2 | 2 | 3 | 2 |
| IDN/CDC184/05 | 2 | 2 | 2 | 2 | 2 | 2 | 2 | 2 |
| IDN/CDC194P/05 | 2 | 2 | 2 | 2 | 2 |  | 2 | 2 |
| IDN/CDC287E/05 | 2 | 3 | 2 | 2 | 2 | 2 | 3 | 2 |
| IDN/CDC292T/05 | 2 | 3 | 2 | 2 | 2 | 2 | 3 | 2 |
| IDN/CDC326/06 | 2 | 3 | 2 | 2 | 2 | 2 | 3 | 2 |
| IDN/CDC329/06 | 2 | 3 | 2 | 2 | 2 | 2 | 3 | 2 |
| IDN/CDC357/06 | 2 | 3 | 2 | 2 | 2 | 2 | 3 | 2 |
| IDN/CDC370/06 | 2 | 2 | 2 | 2 | 2 | 2 | 2 | 2 |
| IDN/CDC390/06 | 2 | 2 | 2 | 2 | 2 | 2 | 2 | 2 |
| IDN/CDC523/06 | 2 | 3 | 2 | 2 | 2 | 2 | 3 | 2 |
| IDN/CDC582/06 | 2 | 3 | 2 | 2 | 2 | 2 | 3 | 2 |
| IDN/CDC594/06 | 1 | 1 | 1 | 1 | 1 | 1 | 1 | 1 |
| IDN/CDC595/06 | 1 | 1 | 1 | 1 | 1 | 1 | 1 | 1 |
| IDN/CDC596/06 | 1 | 1 | 1 | 1 | 1 | 1 | 1 | 1 |
| IDN/CDC597/06 | 1 | 1 | 1 | 1 | 1 | 1 | 1 | 1 |
| IDN/CDC599/06 | 1 | 1 | 1 | 1 | 1 | 1 | 1 | 1 |
| IDN/CDC610/06 | 2 | 3 | 2 | 2 | 2 | 2 | 3 | 2 |
| IDN/CDC623/06 | 2 | 2 | 2 | 2 | 2 | 2 | 2 | 2 |
| IDN/CDC624/06 | 2 | 2 | 2 | 2 | 2 | 2 | 2 | 2 |
| IDN/CDC625/06 | 1 | 1 | 1 | 1 | 1 | 1 | 1 | 1 |
| IDN/CDC634/06 | 2 | 3 | 2 | 2 | 2 | 2 | 3 | 2 |
| IDN/CDC644/06 | 2 | 3 | 2 | 2 | 2 | 2 | 3 | 2 |
| IDN/CDC669/06 | 2 | 3 | 2 | 2 | 2 | 2 | 3 | 2 |
| IDN/CDC699/06 | 2 | 3 | 2 | 2 | 2 | 2 | 3 | 2 |
| IDN/CDC7/05 | 2 | 2 | 2 | 2 | 2 | 2 | 2 | 2 |
| IDN/CDC739/06 | 2 | 3 | 2 | 2 | 2 | 2 | 3 | 2 |
| IDN/CDC742/06 | 2 |  | 2 | 2 | 2 | 2 | 3 | 2 |
| IDN/CDC759/06 | 2 | 3 | 2 | 2 | 2 | 2 | 3 | 2 |
| IDN/CDC835/06 | 2 | 3 | 2 | 2 | 2 | 2 | 3 | 2 |
| IDN/CDC836/06 | 2 | 3 | 2 | 2 | 2 | 2 | 3 | 2 |
| IDN/CDC887/06 | 2 | 3 | 2 | 2 | 2 | 2 | 3 | 2 |
| IDN/CDC938/06 | 2 | 3 | 2 | 2 | 2 | 2 | 3 | 2 |
| IDN/CDC940/06 | 2 | 3 | 2 | 2 | 2 | 2 | 3 | 2 |
| MDk/IDN/Kedri1631 24/06 |  |  | 2 |  | 2 |  |  |  |
| MDk/Jakarta/HABWIN/06 |  |  |  |  | 2 |  |  |  |
| Pg/IDN/Rohkit1631 6/06  (Pg/IDN/Rokhit1631 6/06) |  |  | 2 |  | 2 |  |  |  |
| Qa/Boyolali/BPPV4/04 | X | X | X | X | X | X | X |  |
| Qa/Central Java/SMRG/06 |  |  |  |  | 3 |  |  |  |
| Qa/IDN/Sleman1631 25/06 |  |  | 2 |  |  |  |  |  |
| Qa/Jakarta/JU1/06 |  |  |  |  | 2 |  |  |  |
| Qa/Tasikmalaya/BPPV4/04 | X | X | X | X | X | X | X | X |
| Qa/Yogjakarta/BBVet IX/04 | X | X | X | X | X | X | X | 2 |
| Swan/IDN/Magelang1631 57/07 |  |  | 2 |  |  |  |  |  |
| Swan/IDN/Malang1631 61/07 |  |  |  |  | 2 |  |  |  |
| Tk/Kedaton/BPPV3/04 | X | X | X | X | X | X | X | X |
| Tk/Langkat/BBPV1/05  (Tk/Langkat/BBPVI/05) |  |  | 1 |  | 1 |  |  |  |
|  |  |  |  |  |  |  |  |  |

**End of Table S6**
