# Supplementary material for: Evolutionary and Transmission Dynamics of Reassortant H5N1 Influenza Virus in Indonesia
Source: PLoS Pathog. 2008 Aug 22;4(8):e1000130. doi: 10.1371/journal.ppat.1000130 (PMC2515348; doi:10.1371/journal.ppat.1000130)
Supplement: Table S7 — Accession numbers of the sequences used in this study. (0.93 MB DOC) [file ppat.1000130.s016.doc]

**Table S7**. Accession numbers of the sequences used in this study.

| **Accession #** | **Strain name** | **Accession #** | **Strain name** | **Accession #** | **Strain name** |
| --- | --- | --- | --- | --- | --- |
| **PB2**^a^ |  | **PB1** ^a^ |  | **PA** ^a^ |  |
| AY651738 | A/Ck/HK/YU324/2003 | AY651684 | A/Ck/HK/YU324/2003 | AY651630 | A/Ck/HK/YU324/2003 |
| CY014325 | IDN/CDC287E/05 | CY014324 | IDN/CDC287E/05 | CY014326 | IDN/CDC287E/05 |
| AY651705 | Ck/IDN/PA/03 | DQ493349 | Qa/Yogjakarta/BBVet_IX/04 | AY651601 | Ck/IDN/BL/03 |
| AY651707 | Ck/IDN/BL/03 | AY651653 | Ck/IDN/PA/03 | AY651602 | Ck/IDN/PA/03 |
| AY651710 | Ck/IDN/2A/03 | AY651655 | Ck/IDN/2A/03 | AY651599 | Ck/IDN/2A/03 |
| DQ320865 | Ck/Wonosobo/BPPV4/03 | AY651651 | Ck/IDN/BL/03 | DQ493258 | Ck/Pekalongan/BPPV4/03 |
| DQ492822 | Ck/Sragen/BPPV4/03 | DQ321324 | Ck/Wonosobo/BPPV4/03 | DQ493259 | Ck/Sragen/BPPV4/03 |
| AY651708 | Ck/IDN/5/04 | DQ493345 | Ck/Pekalongan/BPPV4/03 | DQ321258 | Ck/Wonosobo/BPPV4/03 |
| AY651709 | Ck/IDN/4/04 | DQ493346 | Ck/Sragen/BPPV4/03 | AY651600 | Dk/IDN/MS/04 |
| AY651706 | Dk/IDN/MS/04 | AY651652 | Dk/IDN/MS/04 | AY651597 | Ck/IDN/4/04 |
| DQ492825 | Qa/Yogjakarta/BBVet_IX/04 | AY651656 | Ck/IDN/5/04 | AY651598 | Ck/IDN/5/04 |
| DQ492826 | Ck/Kulon_Progo/BBVet_XII_2/04 | AY651654 | Ck/IDN/4/04 | DQ321259 | Ck/Yogjakarta/BBVet_IX/04 |
| DQ492818 | Ck/Malang/BBVet_IV/04 | DQ493347 | Qa/Boyolali/BPPV4/04 | DQ321260 | Ck/Kulon_Progo/BBVet_XII_1/04 |
| DQ492835 | Ck/Kupang_2_NTT/BPPV6/04 | DQ493350 | Ck/Kulon_Progo/BBVet_XII_2/04 | DQ493260 | Qa/Boyolali/BPPV4/04 |
| DQ492836 | Ck/Kupang_3_NTT/BPPV6/04 | DQ493360 | Ck/Kupang_2_NTT/BPPV6/04 | DQ493262 | Qa/Yogjakarta/BBVet_IX/04 |
| DQ492837 | Ck/Kupang_1_NTT/BPPV6/04 | DQ493361 | Ck/Kupang_3_NTT/BPPV6/04 | DQ493263 | Ck/Kulon_Progo/BBVet_XII_2/04 |
| DQ492839 | Tk/Kedaton/BPPV3/04 | DQ493362 | Ck/Kupang_1_NTT/BPPV6/04 | DQ493255 | Ck/Malang/BBVet_IV/04 |
| DQ492829 | Ck/Purwakarta/BBVet_IV/04 | DQ493363 | Ck/Pangkalpinang/BPPV3/04 | DQ493257 | Ck/Ngawi/BPPV4/04 |
| DQ492830 | Ck/Bangli_Bali/BBPV6_1/04 | DQ493364 | Tk/Kedaton/BPPV3/04 | DQ493266 | Ck/Purwakarta/BBVet_IV/04 |
| DQ492831 | Ck/Bangli_Bali/BPPV6_2/04 | DQ493342 | Ck/Malang/BBVet_IV/04 | DQ493267 | Qa/Tasikmalaya/BPPV4/04 |
| DQ492833 | Ck/Mangarai_NTT/BPPV6/04 | DQ493353 | Ck/Purwakarta/BBVet_IV/04 | DQ493268 | Ck/Bangli_Bali/BPPV6_2/04 |
| DQ492845 | Qa/Tasikmalaya/BPPV4/04 | DQ493354 | Qa/Tasikmalaya/BPPV4/04 | DQ493269 | Ck/Bangli_Bali/BBPV6_1/04 |
| DQ492820 | Ck/Ngawi/BPPV4/04 | DQ493355 | Ck/Bangli_Bali/BBPV6_1/04 | DQ493270 | Ck/Jembrana/BPPV6/04 |
| DQ320866 | Ck/Yogjakarta/BBVet_IX/04 | DQ493356 | Ck/Bangli_Bali/BPPV6_2/04 | DQ493271 | Ck/Mangarai_NTT/BPPV6/04 |
| DQ320867 | Ck/Kulon_Progo/BBVet_XII_1/04 | DQ493357 | Ck/Jembrana/BPPV6/04 | DQ493273 | Ck/Kupang_2_NTT/BPPV6/04 |
| DQ320868 | Ck/Bantul/BBVet_I/05 | DQ493358 | Ck/Mangarai_NTT/BPPV6/04 | DQ493274 | Ck/Kupang_3_NTT/BPPV6/04 |
| DQ320869 | Ck/Wajo/BBVM/05 | DQ493344 | Ck/Ngawi/BPPV4/04 | DQ493275 | Ck/Kupang_1_NTT/BPPV6/04 |
| DQ492834 | Dk/Parepare/BBVM/05 | DQ321325 | Ck/Yogjakarta/BBVet_IX/04 | DQ493276 | Ck/Pangkalpinang/BPPV3/04 |
| DQ492840 | Ck/Simalanggang/BPPVI/05 | DQ321326 | Ck/Kulon_Progo/BBVet_XII_1/04 | DQ493277 | Tk/Kedaton/BPPV3/04 |
| DQ492841 | Ck/Tebing_Tinggi/BPPVI/05 | DQ321327 | Ck/Bantul/BBVet_I/05 | DQ493278 | Ck/Simalanggang/BPPVI/05 |
| DQ492842 | Ck/Dairi/BPPVI/05 | DQ321328 | Ck/Wajo/BBVM/05 | DQ493279 | Ck/Tebing_Tinggi/BPPVI/05 |
| DQ492843 | Ck/Deli_Serdang/BPPVI/05 | DQ321323 | Ck/Salatiga/BBVet_I/05 | DQ493280 | Ck/Dairi/BPPVI/05 |
| DQ492844 | Ck/Tarutung/BPPVI/05 | DQ493359 | Dk/Parepare/BBVM/05 | DQ493281 | Ck/Deli_Serdang/BPPVI/05 |
| DQ492819 | Ck/Magetan/BBVW/05 | DQ493343 | Ck/Magetan/BBVW/05 | DQ493282 | Ck/Tarutung/BPPVI/05 |
| DQ492827 | Ck/Gunung_Kidal/BBVW/05 | DQ493365 | Ck/Simalanggang/BPPVI/05 | DQ493272 | Dk/Parepare/BBVM/05 |
| DQ492828 | Ck/Kulon_Progo/BBVW/05 | DQ493366 | Ck/Tebing_Tinggi/BPPVI/05 | DQ493256 | Ck/Magetan/BBVW/05 |
| DQ492824 | Ck/Purworejo/BBVW/05 | DQ493367 | Ck/Dairi/BPPVI/05 | DQ493264 | Ck/Gunung_Kidal/BBVW/05 |
| DQ320864 | Ck/Salatiga/BBVet_I/05 | DQ493368 | Ck/Deli_Serdang/BPPVI/05 | DQ493265 | Ck/Kulon_Progo/BBVW/05 |
| CY014167 | IDN/5/05 | DQ493369 | Ck/Tarutung/BPPVI/05 | DQ493261 | Ck/Purworejo/BBVW/05 |
| CY014175 | IDN/CDC7/05 | DQ493351 | Ck/Gunung_Kidal/BBVW/05 | DQ321262 | Ck/Wajo/BBVM/05 |
| CY014182 | Ck/IDN/CDC25/05 | DQ493352 | Ck/Kulon_Progo/BBVW/05 | DQ321257 | Ck/Salatiga/BBVet_I/05 |
| CY014190 | Ck/IDN/CDC24/05 | DQ493348 | Ck/Purworejo/BBVW/05 | CY014171 | IDN/5/05 |
| CY014322 | IDN/CDC194P/05 | CY014170 | IDN/5/05 | CY014169 | IDN/CDC7/05 |
| CY014320 | IDN/CDC184/05 | CY014176 | IDN/CDC7/05 | CY014184 | Ck/IDN/CDC25/05 |
| CY014333 | IDN/CDC292T/05 | CY014191 | Ck/IDN/CDC24/05 | CY014192 | Ck/IDN/CDC24/05 |
| CY017683 | IDN/CDC759/06 | CY014183 | Ck/IDN/CDC25/05 | CY014321 | IDN/CDC184/05 |
| CY014348 | IDN/CDC329/06 | CY014323 | IDN/CDC194P/05 | CY014334 | IDN/CDC292T/05 |
| CY014337 | IDN/CDC326/06 | CY014319 | IDN/CDC184/05 | CY014542 | IDN/CDC759/06 |
| CY014317 | Ct/IDN/CDC1/06 | CY014335 | IDN/CDC292T/05 | CY014350 | IDN/CDC329/06 |
| CY014352 | IDN/CDC357/06 | CY017684 | IDN/CDC759/06 | CY014338 | IDN/CDC326/06 |
| CY014356 | IDN/CDC370/06 | CY014349 | IDN/CDC329/06 | CY014318 | Ct/IDN/CDC1/06 |
| CY014367 | IDN/CDC390/06 | CY014336 | IDN/CDC326/06 | CY014353 | IDN/CDC357/06 |
| CY014308 | IDN/CDC523/06 | CY014316 | Ct/IDN/CDC1/06 | CY014354 | IDN/CDC370/06 |
| CY014391 | IDN/CDC582/06 | CY014351 | IDN/CDC357/06 | CY014366 | IDN/CDC390/06 |
| CY014293 | IDN/CDC597/06 | CY014355 | IDN/CDC370/06 | CY014310 | IDN/CDC523/06 |
| CY014285 | IDN/CDC596/06 | CY014365 | IDN/CDC390/06 | CY014389 | IDN/CDC582/06 |
| CY014392 | IDN/CDC599/06 | CY014309 | IDN/CDC523/06 | CY014295 | IDN/CDC597/06 |
| CY014396 | IDN/CDC610/06 | CY014390 | IDN/CDC582/06 | CY014287 | IDN/CDC596/06 |
| CY014404 | IDN/CDC623/06 | CY014294 | IDN/CDC597/06 | CY014302 | IDN/CDC599/06 |
| CY014420 | IDN/CDC624/06 | CY014286 | IDN/CDC596/06 | CY014394 | IDN/CDC610/06 |
| CY014436 | IDN/CDC625/06 | CY014301 | IDN/CDC599/06 | CY014402 | IDN/CDC623/06 |
| CY014444 | IDN/CDC634/06 | CY014395 | IDN/CDC610/06 | CY014418 | IDN/CDC624/06 |
| CY014520 | IDN/CDC644/06 | CY014403 | IDN/CDC623/06 | CY014434 | IDN/CDC625/06 |
| CY014277 | IDN/CDC595/06 | CY014419 | IDN/CDC624/06 | CY014442 | IDN/CDC634/06 |
| CY014269 | IDN/CDC594/06 | CY014435 | IDN/CDC625/06 | CY014519 | IDN/CDC644/06 |
| CY014484 | IDN/CDC669/06 | CY014443 | IDN/CDC634/06 | CY014279 | IDN/CDC595/06 |
| CY014500 | IDN/CDC699/06 | CY014513 | IDN/CDC644/06 | CY014271 | IDN/CDC594/06 |
| CY014526 | IDN/CDC739/06 | CY014278 | IDN/CDC595/06 | CY014482 | IDN/CDC669/06 |
| CY014534 | IDN/CDC742/06 | CY014270 | IDN/CDC594/06 | CY014498 | IDN/CDC699/06 |
| CY017659 | IDN/CDC835/06 | CY014483 | IDN/CDC669/06 | CY014528 | IDN/CDC739/06 |
| CY017667 | IDN/CDC836/06 | CY014499 | IDN/CDC699/06 | CY014536 | IDN/CDC742/06 |
| CY017685 | IDN/CDC887/06 | CY014527 | IDN/CDC739/06 | CY017661 | IDN/CDC835/06 |
| CY017635 | IDN/CDC938/06 | CY014535 | IDN/CDC742/06 | CY017669 | IDN/CDC836/06 |
| CY017651 | IDN/CDC940/06 | CY017660 | IDN/CDC835/06 | CY017687 | IDN/CDC887/06 |
| CY019405 | IDN/CDC1046/07 | CY017668 | IDN/CDC836/06 | CY017637 | IDN/CDC938/06 |
| CY019421 | IDN/CDC1047/07 | CY017686 | IDN/CDC887/06 | CY017653 | IDN/CDC940/06 |
| CY019349 | IDN/CDC1031/07 | CY017636 | IDN/CDC938/06 | CY019407 | IDN/CDC1046/07 |
| CY019381 | IDN/CDC1032/07 | CY017652 | IDN/CDC940/06 | CY019423 | IDN/CDC1047/07 |
| ISDN128033 | IDN/6/05 | CY019406 | IDN/CDC1046/07 | CY019351 | IDN/CDC1031/07 |
| **HA** ^a^ |  | CY019422 | IDN/CDC1047/07 | CY019383 | IDN/CDC1032/07 |
| AY651358 | A/Ck/HK/YU324/2003 | CY019350 | IDN/CDC1031/07 | ISDN136846 | IDN/298H/06 |
| CY014198 | IDN/CDC287E/05 | CY019382 | IDN/CDC1032/07 | ISDN128031 | IDN/6/05 |
| AY651323 | Ck/IDN/2A/03 | ISDN136847 | IDN/298H/06 | ISDN130917 | IDN/195H/05 |
| AY651320 | Ck/IDN/PA/03 | ISDN130918 | IDN/195H/05 | **NA** ^a^ |  |
| AY651321 | Ck/IDN/BL/03 | ISDN125789 | IDN/5/05 | AY651465 | A/Ck/HK/YU324/2003 |
| DQ320929 | Ck/Wonosobo/BPPV4/03 | ISDN128032 | IDN/6/05 | AY651437 | Ck/IDN/4/04 |
| DQ497645 | Ck/Pekalongan/BPPV4/03 | **NP** ^a^ |  | DQ493006 | Ck/Jembrana/BPPV6/04 |
| DQ497646 | Ck/Sragen/BPPV4/03 | AY651520 | A/Ck/HK/YU324/2003 | DQ492993 | Ck/Ngawi/BPPV4/04 |
| EF473080 | Ck/IDN/7/03 | DQ493101 | Tk/Kedaton/BPPV3/04 | DQ493012 | Ck/Pangkalpinang/BPPV3/04 |
| EF473081 | Ck/IDN/11/03 | AY651485 | Ck/IDN/BL/03 | DQ492994 | Ck/Pekalongan/BPPV4/03 |
| AY651322 | Dk/IDN/MS/04 | AY651487 | Ck/IDN/PA/03 | DQ492995 | Ck/Sragen/BPPV4/03 |
| AY651324 | Ck/IDN/4/04 | AY651490 | Ck/IDN/2A/03 | DQ321060 | Ck/Wonosobo/BPPV4/03 |
| AY651325 | Ck/IDN/5/04 | DQ321130 | Ck/Wonosobo/BPPV4/03 | DQ492996 | Qa/Boyolali/BPPV4/04 |
| DQ497647 | Qa/Boyolali/BPPV4/04 | DQ493082 | Ck/Pekalongan/BPPV4/03 | AY651435 | Ck/IDN/2A/03 |
| DQ497649 | Qa/Yogjakarta/BBVet_IX/04 | DQ493083 | Ck/Sragen/BPPV4/03 | AY651432 | Ck/IDN/BL/03 |
| DQ497650 | Ck/Kulon_Progo/BBVet_XII_2/04 | AY651488 | Ck/IDN/4/04 | AY651433 | Ck/IDN/PA/03 |
| DQ497653 | Ck/Purwakarta/BBVet_IV/04 | AY651486 | Dk/IDN/MS/04 | AM183681 | Ck/IDN/R134/03 |
| DQ497654 | Qa/Tasikmalaya/BPPV4/04 | AY651489 | Ck/IDN/5/04 | EF473082 | Ck/IDN/7/03 |
| DQ497655 | Ck/Bangli_Bali/BBPV6_1/04 | DQ493084 | Qa/Boyolali/BPPV4/04 | EF473083 | Ck/IDN/11/03 |
| DQ497656 | Ck/Bangli_Bali/BPPV6_2/04 | DQ493079 | Ck/Malang/BBVet_IV/04 | AY651434 | Dk/IDN/MS/04 |
| DQ497657 | Ck/Jembrana/BPPV6/04 | DQ493086 | Qa/Yogjakarta/BBVet_IX/04 | AY651436 | Ck/IDN/5/04 |
| DQ497658 | Ck/Mangarai_NTT/BPPV6/04 | DQ493087 | Ck/Kulon_Progo/BBVet_XII_2/04 | DQ493018 | Tk/Kedaton/BPPV3/04 |
| DQ497644 | Ck/Ngawi/BPPV4/04 | DQ493090 | Ck/Purwakarta/BBVet_IV/04 | DQ321061 | Ck/Yogjakarta/BBVet_IX/04 |
| DQ497660 | Ck/Kupang_2_NTT/BPPV6/04 | DQ493091 | Qa/Tasikmalaya/BPPV4/04 | DQ321062 | Ck/Kulon_Progo/BBVet_XII_1/04 |
| DQ497661 | Ck/Kupang_3_NTT/BPPV6/04 | DQ493092 | Ck/Bangli_Bali/BBPV6_1/04 | DQ492991 | Ck/Malang/BBVet_IV/04 |
| DQ497662 | Ck/Kupang_1_NTT/BPPV6/04 | DQ493093 | Ck/Bangli_Bali/BPPV6_2/04 | DQ492998 | Qa/Yogjakarta/BBVet_IX/04 |
| DQ497663 | Ck/Pangkalpinang/BPPV3/04 | DQ493094 | Ck/Jembrana/BPPV6/04 | DQ492999 | Ck/Kulon_Progo/BBVet_XII_2/04 |
| DQ497664 | Tk/Kedaton/BPPV3/04 | DQ493095 | Ck/Mangarai_NTT/BPPV6/04 | DQ493002 | Ck/Purwakarta/BBVet_IV/04 |
| DQ497642 | Ck/Malang/BBVet_IV/04 | DQ493097 | Ck/Kupang_2_NTT/BPPV6/04 | DQ493003 | Qa/Tasikmalaya/BPPV4/04 |
| DQ320930 | Ck/Yogjakarta/BBVet_IX/04 | DQ493098 | Ck/Kupang_3_NTT/BPPV6/04 | DQ493004 | Ck/Bangli_Bali/BPPV6_2/04 |
| DQ320931 | Ck/Kulon_Progo/BBVet_XII_1/04 | DQ493099 | Ck/Kupang_1_NTT/BPPV6/04 | DQ493005 | Ck/Bangli_Bali/BBPV6_1/04 |
| DQ320932 | Ck/Bantul/BBVet_I/05 | DQ493100 | Ck/Pangkalpinang/BPPV3/04 | DQ493007 | Ck/Mangarai_NTT/BPPV6/04 |
| DQ320933 | Ck/Wajo/BBVM/05 | DQ321126 | Ck/Yogjakarta/BBVet_IX/04 | DQ493009 | Ck/Kupang_2_NTT/BPPV6/04 |
| DQ320928 | Ck/Salatiga/BBVet_I/05 | DQ321127 | Ck/Kulon_Progo/BBVet_XII_1/04 | DQ493010 | Ck/Kupang_3_NTT/BPPV6/04 |
| DQ497643 | Ck/Magetan/BBVW/05 | DQ493081 | Ck/Ngawi/BPPV4/04 | DQ493011 | Ck/Kupang_1_NTT/BPPV6/04 |
| DQ497665 | Ck/Simalanggang/BPPVI/05 | DQ321128 | Ck/Bantul/BBVet_I/05 | DQ493013 | Ck/Simalanggang/BPPVI/05 |
| DQ497666 | Ck/Tebing_Tinggi/BPPVI/05 | DQ321129 | Ck/Wajo/BBVM/05 | DQ493014 | Ck/Tebing_Tinggi/BPPVI/05 |
| DQ497667 | Ck/Dairi/BPPVI/05 | DQ321125 | Ck/Salatiga/BBVet_I/05 | DQ493015 | Ck/Dairi/BPPVI/05 |
| DQ497668 | Ck/Deli_Serdang/BPPVI/05 | DQ493102 | Ck/Simalanggang/BPPVI/05 | DQ493016 | Ck/Deli_Serdang/BPPVI/05 |
| DQ497669 | Ck/Tarutung/BPPVI/05 | DQ493103 | Ck/Tebing_Tinggi/BPPVI/05 | DQ493017 | Ck/Tarutung/BPPVI/05 |
| DQ497659 | Dk/Parepare/BBVM/05 | DQ493104 | Ck/Dairi/BPPVI/05 | DQ493008 | Dk/Parepare/BBVM/05 |
| DQ497651 | Ck/Gunung_Kidal/BBVW/05 | DQ493105 | Ck/Deli_Serdang/BPPVI/05 | DQ493000 | Ck/Gunung_Kidal/BBVW/05 |
| DQ497648 | Ck/Purworejo/BBVW/05 | DQ493106 | Ck/Tarutung/BPPVI/05 | DQ493001 | Ck/Kulon_Progo/BBVW/05 |
| EU124049 | Ck/IDN/Wates1/05 | DQ493096 | Dk/Parepare/BBVM/05 | DQ492992 | Ck/Magetan/BBVW/05 |
| EU124050 | Ck/IDN/Wates130/05 | DQ493088 | Ck/Gunung_Kidal/BBVW/05 | DQ492997 | Ck/Purworejo/BBVW/05 |
| EU124051 | Ck/IDN/Wates77/05 | DQ493089 | Ck/Kulon_Progo/BBVW/05 | DQ321063 | Ck/Bantul/BBVet_I/05 |
| EU124052 | Ck/IDN/Wates80/05 | DQ493080 | Ck/Magetan/BBVW/05 | DQ321064 | Ck/Wajo/BBVM/05 |
| EU124053 | Ck/IDN/Wates126/05 | DQ493085 | Ck/Purworejo/BBVW/05 | DQ321059 | Ck/Salatiga/BBVet_I/05 |
| EU124054 | Ck/IDN/Wates83/05 | CY014172 | IDN/5/05 | EU124055 | Ck/IDN/Wates1/05 |
| EF541394 | IDN/5/05 | CY014178 | IDN/CDC7/05 | EU124056 | Ck/IDN/Wates130/05 |
| EU124090 | Tk/Langkat/BBPVI/05 | CY014186 | Ck/IDN/CDC25/05 | EU124057 | Ck/IDN/Wates77/05 |
| EU124095 | Ck/Agam/BBPVI/05 | CY014252 | IDN/CDC194P/05 | EU124058 | Ck/IDN/Wates80/05 |
| EU124096 | Ck/Salam/BBPV_II/05 | CY014241 | IDN/CDC184/05 | EU124059 | Ck/IDN/Wates126/05 |
| EU124097 | Ck/Murao_Jambi/BBPV_II/05 | CY014251 | IDN/CDC287E/05 | EU124060 | Ck/IDN/Wates83/05 |
| EU124098 | Ck/Duma/BBPV_II/05 | CY014253 | IDN/CDC292T/05 | EU124074 | Dk/IBufeleng/BPPV1/05 |
| EU124099 | Ck/Rokan_Hilli/BPPV_II/05 | CY014415 | IDN/CDC623E/06 | AM183682 | Ck/IDN/R60/05 |
| EU124100 | Ck/Siak/BPPV_II/05 | CY014544 | IDN/CDC759/06 | EU124118 | Ck/Agam/BBPV1/05 |
| EU124101 | Ck/Pakun_Baru/BPPV_II/05 | CY014250 | IDN/CDC329/06 | EU124119 | Ck/Salam/BBPV11/05 |
| EU124102 | Ck/Palembang/BPPV_III/05 | CY014243 | IDN/CDC326/06 | EU124120 | Ck/Murao_Jambi/BPPV11/05 |
| EU124103 | Ck/Sembawa/BPPV_III/05 | CY014254 | Ct/IDN/CDC1/06 | EU124121 | Ck/Duma/BPPV11/05 |
| EU124104 | Dk/Pali/BBVW1358/05 | CY014246 | IDN/CDC357/06 | EU124122 | Ck/Rokan_Hilli/BPPV11/05 |
| EU124105 | Dk/Madiun/BBVW1358/05 | CY014247 | IDN/CDC370/06 | EU124123 | Ck/Siak/BPPV11/05 |
| EU124106 | Dk/Tabanan/BPPV1/05 | CY014249 | IDN/CDC390/06 | EU124124 | Ck/Pakunbaru/BPPV11/05 |
| EU124107 | Dk/Bufeleng/BPPV1/05 | CY014312 | IDN/CDC523/06 | EU124127 | Ck/Palembang/BPPV111/05 |
| EU124108 | Ck/Deli_Serdang/BPPV1/05 | CY014387 | IDN/CDC582/06 | EU124131 | Dk/Pali/BBVW/05 |
| EU124147 | Ck/Madiun/BBVW1420/05 | CY014297 | IDN/CDC597/06 | EU124132 | Dk/Madiun/BBVW1358/05 |
| AM183670 | Ck/IDN/R60/05 | CY014289 | IDN/CDC596/06 | EF541395 | IDN/5/05 |
| EU124094 | Ck/Medan/BPPV1_498/05 | CY014304 | IDN/CDC599/06 | EU124116 | Ck/Pidie/BBPV1/05 |
| EU124093 | Ck/Pidie/BPPV1/05 | CY014399 | IDN/CDC610/06 | EU124117 | Ck/Medan/BBPV1_498/05 |
| EU124092 | Ck/Medan/BPPV1_534/05 | CY014407 | IDN/CDC623/06 | EU124115 | Ck/Medan/BBPV1_534/05 |
| EU124091 | Ck/Deli_Derdang/BBPVI/05 | CY014423 | IDN/CDC624/06 | EU124114 | Ck/Deli_Derdang/BBPV1/05 |
| CY014177 | IDN/CDC7/05 | CY014439 | IDN/CDC625/06 | CY014179 | IDN/CDC7/05 |
| EU124080 | Ck/Medan/BBPV1_571/05 | CY014447 | IDN/CDC634/06 | EU124113 | Tk/Langkat/BBPV1/05 |
| EU124081 | Ck/Medan/BBPV1_576/05 | CY014516 | IDN/CDC644/06 | EU124112 | Ck/Medan/BBPV1_576/05 |
| EU124082 | Ck/Langkat/BBPV1_576/05 | CY014281 | IDN/CDC595/06 | EU124111 | Ck/Langkat/BBPV1/05 |
| CY014193 | Ck/IDN/CDC24/05 | CY014273 | IDN/CDC594/06 | CY014187 | Ck/IDN/CDC25/05 |
| CY014185 | Ck/IDN/CDC25/05 | CY014487 | IDN/CDC669/06 | CY014194 | Ck/IDN/CDC24/05 |
| CY014168 | IDN/CDC194P/05 | CY014503 | IDN/CDC699/06 | CY014240 | IDN/CDC194P/05 |
| EU124083 | Ck/Taput/BBPV1_576/05 | CY014530 | IDN/CDC739/06 | EU124110 | Ck/Taput/BBPV1/05 |
| CY014197 | IDN/CDC184/05 | CY014538 | IDN/CDC742/06 | CY014237 | IDN/CDC184/05 |
| CY014201 | IDN/CDC292T/05 | CY017663 | IDN/CDC835/06 | CY014238 | IDN/CDC287E/05 |
| CY014543 | IDN/CDC759/06 | CY017671 | IDN/CDC836/06 | CY014228 | IDN/CDC292T/05 |
| EU124084 | Dk/Indramayu/BBPW109/06 | CY017689 | IDN/CDC887/06 | CY014545 | IDN/CDC759/06 |
| EU124085 | Ck/Bandar_Lampung/BBPVIII/06 | CY017639 | IDN/CDC938/06 | EU124133 | Dk/Indramayu/BBVW109/06 |
| EU124086 | Ck/Way_Kanan/BBPVIII/06 | CY017655 | IDN/CDC940/06 | EU124129 | Ck/Way_Kanan/BPPV111/06 |
| EU124087 | Ck/Pulau_Rampang/BBPVII/06 | CY019409 | IDN/CDC1046/07 | EU124130 | Ck/Bandar_Lampung/BPPV111/06 |
| EU124088 | Ck/Padang/BBPVII/06 | CY019425 | IDN/CDC1047/07 | EU124125 | Ck/Padang/BPPV11/06 |
| EU124148 | Ck/West_Java/PWT_WIJ/06 | CY019353 | IDN/CDC1031/07 | EU124126 | Ck/Paulau_Rampang/BPPV11/06 |
| EU124146 | Ck/Gunung_Kidul/BBVW/06 | CY019385 | IDN/CDC1032/07 | EU124216 | Ck/IDN/Padang1631_1/06 |
| EU124150 | Ck/West_Java/SMI_CSLK_EC/06 | ISDN136844 | IDN/298H/06 | EU124217 | Ck/IDN/Siak1631_2/06 |
| EU124196 | Ck/IDN/Padang1631_1/06 | ISDN130915 | IDN/195H/05 | EU124109 | Ck/Karo/BBPV1/06 |
| EU124197 | Ck/IDN/Siak1631_2/06 | ISDN128029 | IDN/6/05 | CY014235 | IDN/CDC329/06 |
| EU124089 | Ck/Karo/BBPVII/06 | **M1** ^a^ |  | CY014239 | IDN/CDC326/06 |
| CY014206 | IDN/CDC329/06 | AY651407 | A/Ck/HK/YU324/2003 | CY014234 | Ct/IDN/CDC1/06 |
| CY014204 | IDN/CDC326/06 | AY651374 | Ck/IDN/BL/03 | CY014230 | IDN/CDC357/06 |
| CY014208 | Ct/IDN/CDC1/06 | AY651376 | Ck/IDN/PA/03 | CY014231 | IDN/CDC370/06 |
| CY014207 | IDN/CDC357/06 | AY651377 | Ck/IDN/2A/03 | CY014233 | IDN/CDC390/06 |
| CY014209 | IDN/CDC370/06 | DQ320995 | Ck/Wonosobo/BPPV4/03 | CY014313 | IDN/CDC523/06 |
| CY014213 | IDN/CDC390/06 | DQ492906 | Ck/Pekalongan/BPPV4/03 | CY014386 | IDN/CDC582/06 |
| CY014311 | IDN/CDC523/06 | DQ492907 | Ck/Sragen/BPPV4/03 | EU124226 | Muscovy_Dk/IDN/Kedri1631_24/06 |
| CY014384 | IDN/CDC582/06 | EF473084 | Ck/IDN/11/03 | CY014298 | IDN/CDC597/06 |
| EU124160 | Ck/West_Java/SMI_PAT/06 | EF473079 | Ck/IDN/7/03 | CY014290 | IDN/CDC596/06 |
| EU124161 | Ck/West_Java/SMI_ENDRI2/06 | AY651378 | Ck/IDN/4/04 | CY014305 | IDN/CDC599/06 |
| EU124162 | Ck/West_Java/SMI_ENDRI1/06 | AY651379 | Ck/IDN/5/04 | CY014398 | IDN/CDC610/06 |
| EU124163 | Ck/West_Java/HAMD/06 | AY651375 | Dk/IDN/MS/04 | CY014406 | IDN/CDC623/06 |
| EU124206 | Muscovy_Dk/IDN/Kedri1631_24/06 | DQ492908 | Qa/Boyolali/BPPV4/04 | CY014422 | IDN/CDC624/06 |
| CY014296 | IDN/CDC597/06 | DQ492913 | Ck/Purwakarta/BBVet_IV/04 | CY014438 | IDN/CDC625/06 |
| CY014288 | IDN/CDC596/06 | DQ492914 | Qa/Tasikmalaya/BPPV4/04 | CY014446 | IDN/CDC634/06 |
| CY014303 | IDN/CDC599/06 | DQ492915 | Ck/Bangli_Bali/BPPV6_2/04 | CY014515 | IDN/CDC644/06 |
| CY014393 | IDN/CDC610/06 | DQ492916 | Ck/Bangli_Bali/BBPV6_1/04 | CY014282 | IDN/CDC595/06 |
| CY014401 | IDN/CDC623/06 | DQ492917 | Ck/Jembrana/BPPV6/04 | CY014274 | IDN/CDC594/06 |
| CY014417 | IDN/CDC624/06 | DQ492918 | Ck/Mangarai_NTT/BPPV6/04 | EU124227 | Qa/IDN/Sleman1631_25/06 |
| CY014433 | IDN/CDC625/06 | DQ492920 | Ck/Kupang_2_NTT/BPPV6/04 | EU124218 | Ck/IDN/Agam1631_3/06 |
| CY014441 | IDN/CDC634/06 | DQ492921 | Ck/Kupang_3_NTT/BPPV6/04 | CY014486 | IDN/CDC669/06 |
| CY014518 | IDN/CDC644/06 | DQ492922 | Ck/Kupang_1_NTT/BPPV6/04 | EU124224 | Ck/IDN/Rejang_Lebong1631_22/06 |
| CY014280 | IDN/CDC595/06 | DQ492923 | Ck/Pangkalpinang/BPPV3/04 | EU124228 | Ck/IDN/Gunung_Kidul1631_33/06 |
| CY014272 | IDN/CDC594/06 | DQ492924 | Tk/Kedaton/BPPV3/04 | CY014502 | IDN/CDC699/06 |
| EU124198 | Ck/IDN/Agam1631_3/06 | DQ492910 | Qa/Yogjakarta/BBVet_IX/04 | CY014531 | IDN/CDC739/06 |
| EU124159 | Qa/Central_Java/SMRG/06 | DQ492930 | Ck/Kulon_Progo/BBVet_XII_2/04 | CY014539 | IDN/CDC742/06 |
| CY014481 | IDN/CDC669/06 | DQ320996 | Ck/Yogjakarta/BBVet_IX/04 | EU124225 | Ck/IDN/Lampung1631_23/06 |
| EU124204 | Ck/IDN/Rejang_Lebong1631_22/06 | DQ320997 | Ck/Kulon_Progo/BBVet_XII_1/04 | EU124222 | Ck/IDN/Bangka_Seletan1631_20/06 |
| EU124208 | Ck/IDN/Gunung_Kidul1631_33/06 | DQ492905 | Ck/Ngawi/BPPV4/04 | EU124223 | Ck/IDN/Bangka_Seletan1631_21/06 |
| EU124154 | Ck/West_Java/TASIK2/06 | DQ492903 | Ck/Malang/BBVet_IV/04 | CY017664 | IDN/CDC835/06 |
| EU124155 | Ck/West_Java/TASIK1/06 | DQ492904 | Ck/Magetan/BBVW/05 | CY017672 | IDN/CDC836/06 |
| EU124156 | Ck/Papua/TB15/06 | DQ320998 | Ck/Bantul/BBVet_I/05 | EU124219 | Pg/IDN/Rohkit1631_6/06 |
| EU124157 | Ck/Papua/TB1/06 | DQ320999 | Ck/Wajo/BBVM/05 | EU124229 | Ck/IDN/Kulon1631_47/06 |
| EU124158 | Ck/Papua/TA5/06 | DQ320994 | Ck/Salatiga/BBVet_I/05 | CY017690 | IDN/CDC887/06 |
| CY014497 | IDN/CDC699/06 | DQ492911 | Ck/Gunung_Kidal/BBVW/05 | EU124230 | Ck/IDN/Bandung1631_49/06 |
| EU124151 | Ck/West_Java/TASIKSOL/06 | DQ492912 | Ck/Kulon_Progo/BBVW/05 | EU124231 | Ck/IDN/Garut1631_51/06 |
| EU124152 | Qa/Jakarta/JU1/06 | DQ492925 | Ck/Simalanggang/BPPVI/05 | CY017640 | IDN/CDC938/06 |
| EU124153 | Ck/West_Java/GARUT_MAY/06 | DQ492926 | Ck/Tebing_Tinggi/BPPVI/05 | CY017656 | IDN/CDC940/06 |
| EU124277 | Ck/West_Java/TASIKSOB/06 | DQ492927 | Ck/Dairi/BPPVI/05 | EU124220 | Ck/IDN/Pekenbaru1631_11/06 |
| CY014529 | IDN/CDC739/06 | DQ492928 | Ck/Deli_Serdang/BPPVI/05 | EU124221 | Ck/IDN/Belitung_Timor1631_18/06 |
| CY014537 | IDN/CDC742/06 | DQ492929 | Ck/Tarutung/BPPVI/05 | EU124232 | Ck/IDN/Magelang1631_57/07 |
| EU124276 | Ck/West_Java/SMI_CSLK_EB/06 | DQ492919 | Dk/Parepare/BBVM/05 | CY019410 | IDN/CDC1046/07 |
| EU124205 | Ck/IDN/Lampung1631_23/06 | DQ492909 | Ck/Purworejo/BBVW/05 | CY019426 | IDN/CDC1047/07 |
| EU124202 | Ck/IDN/Bangka_Seletan1631_20/06 | CY014173 | IDN/5/05 | CY019354 | IDN/CDC1031/07 |
| EU124203 | Ck/IDN/Bangka_Seletan1631_21/06 | CY014180 | IDN/CDC7/05 | CY019386 | IDN/CDC1032/07 |
| EU124149 | Muscovy_Dk/Jakarta/HABWIN/06 | CY014195 | Ck/IDN/CDC24/05 | EU124233 | Swan/IDN/Magelang1631_57/07 |
| CY017662 | IDN/CDC835/06 | CY014188 | Ck/IDN/CDC25/05 | EU124234 | Ck/IDN/Semerang1631_62/07 |
| CY017670 | IDN/CDC836/06 | CY014225 | IDN/CDC194P/05 | ISDN136843 | IDN/298H/06 |
| EU124199 | Pg/IDN/Rokhit1631_6/06 | CY014214 | IDN/CDC184/05 | ISDN128027 | IDN/6/05 |
| EU124209 | Ck/IDN/Kulon1631_47/06 | CY014226 | IDN/CDC287E/05 | ISDN130914 | IDN/195H/05 |
| CY017688 | IDN/CDC887/06 | CY014215 | IDN/CDC292T/05 | **NS** ^a^ |  |
| EU124210 | Ck/IDN/Bandung1631_49/06 | CY014546 | IDN/CDC759/06 | AY651572 | A/Ck/HK/YU324/2003 |
| EU124211 | Ck/IDN/Garut1631_51/06 | CY014219 | IDN/CDC329/06 | DQ493179 | Qa/Tasikmalaya/BPPV4/04 |
| CY017638 | IDN/CDC938/06 | CY014216 | IDN/CDC326/06 | AY651539 | Ck/IDN/BL/03 |
| CY017654 | IDN/CDC940/06 | CY014221 | Ct/IDN/CDC1/06 | AY651541 | Ck/IDN/PA/03 |
| EU124200 | Ck/IDN/Pekenbaru1631_11/06 | CY014220 | IDN/CDC357/06 | AY651543 | Ck/IDN/2A/03 |
| EU124201 | Ck/IDN/Belitung_Timor1631_18/06 | CY014222 | IDN/CDC370/06 | DQ493170 | Ck/Pekalongan/BPPV4/03 |
| EU124212 | Ck/IDN/Magelang1631_57/07 | CY014224 | IDN/CDC390/06 | DQ493171 | Ck/Sragen/BPPV4/03 |
| CY019408 | IDN/CDC1046/07 | CY014314 | IDN/CDC523/06 | DQ321192 | Ck/Wonosobo/BPPV4/03 |
| CY019424 | IDN/CDC1047/07 | CY014385 | IDN/CDC582/06 | AY651544 | Ck/IDN/5/04 |
| CY019352 | IDN/CDC1031/07 | CY014299 | IDN/CDC597/06 | AY651542 | Ck/IDN/4/04 |
| CY019384 | IDN/CDC1032/07 | CY014291 | IDN/CDC596/06 | AY651540 | Dk/IDN/MS/04 |
| EU124213 | Swan/IDN/Malang1631_61/07 | CY014306 | IDN/CDC599/06 | DQ321193 | Ck/Yogjakarta/BBVet_IX/04 |
| EU124215 | Ck/IDN/Soppeng1631_71/07 | CY014397 | IDN/CDC610/06 | DQ321194 | Ck/Kulon_Progo/BBVet_XII_1/04 |
| EU124214 | Ck/IDN/Semerang1631_62/07 | CY014405 | IDN/CDC623/06 | DQ493167 | Ck/Malang/BBVet_IV/04 |
| ISDN136841 | IDN/298H/06 | CY014421 | IDN/CDC624/06 | DQ493172 | Qa/Boyolali/BPPV4/04 |
| ISDN128026 | IDN/6/05 | CY014437 | IDN/CDC625/06 | DQ493174 | Qa/Yogjakarta/BBVet_IX/04 |
| ISDN130912 | IDN/195H/05 | CY014445 | IDN/CDC634/06 | DQ493175 | Ck/Kulon_Progo/BBVet_XII_2/04 |
| **MP** ^a^ |  | CY014514 | IDN/CDC644/06 | DQ493185 | Ck/Kupang_2_NTT/BPPV6/04 |
| AY651407 | A/Ck/HK/YU324/2003 | CY014283 | IDN/CDC595/06 | DQ493186 | Ck/Kupang_3_NTT/BPPV6/04 |
| AY651374 | Ck/IDN/BL/03 | CY014275 | IDN/CDC594/06 | DQ493187 | Ck/Kupang_1_NTT/BPPV6/04 |
| AY651376 | Ck/IDN/PA/03 | CY014485 | IDN/CDC669/06 | DQ493188 | Ck/Pangkalpinang/BPPV3/04 |
| AY651377 | Ck/IDN/2A/03 | CY014501 | IDN/CDC699/06 | DQ493189 | Tk/Kedaton/BPPV3/04 |
| DQ320995 | Ck/Wonosobo/BPPV4/03 | CY014532 | IDN/CDC739/06 | DQ493178 | Ck/Purwakarta/BBVet_IV/04 |
| DQ492906 | Ck/Pekalongan/BPPV4/03 | CY017665 | IDN/CDC835/06 | DQ493180 | Ck/Bangli_Bali/BBPV6_1/04 |
| DQ492907 | Ck/Sragen/BPPV4/03 | CY017673 | IDN/CDC836/06 | DQ493181 | Ck/Bangli_Bali/BPPV6_2/04 |
| EF473084 | Ck/IDN/11/03 | CY017691 | IDN/CDC887/06 | DQ493182 | Ck/Jembrana/BPPV6/04 |
| EF473079 | Ck/IDN/7/03 | CY017641 | IDN/CDC938/06 | DQ493183 | Ck/Mangarai_NTT/BPPV6/04 |
| AY651378 | Ck/IDN/4/04 | CY017657 | IDN/CDC940/06 | DQ493169 | Ck/Ngawi/BPPV4/04 |
| AY651379 | Ck/IDN/5/04 | CY019411 | IDN/CDC1046/07 | DQ493184 | Dk/Parepare/BBVM/05 |
| AY651375 | Dk/IDN/MS/04 | CY019427 | IDN/CDC1047/07 | DQ493190 | Ck/Simalanggang/BPPVI/05 |
| DQ492908 | Qa/Boyolali/BPPV4/04 | CY019355 | IDN/CDC1031/07 | DQ493191 | Ck/Tebing_Tinggi/BPPVI/05 |
| DQ492913 | Ck/Purwakarta/BBVet_IV/04 | CY019387 | IDN/CDC1032/07 | DQ493192 | Ck/Dairi/BPPVI/05 |
| DQ492914 | Qa/Tasikmalaya/BPPV4/04 | ISDN136842 | IDN/298H/06 | DQ493193 | Ck/Deli_Serdang/BPPVI/05 |
| DQ492915 | Ck/Bangli_Bali/BPPV6_2/04 | ISDN128028 | IDN/6/05 | DQ493194 | Ck/Tarutung/BPPVI/05 |
| DQ492916 | Ck/Bangli_Bali/BBPV6_1/04 | ISDN130913 | IDN/195H/05 | DQ493176 | Ck/Gunung_Kidal/BBVW/05 |
| DQ492917 | Ck/Jembrana/BPPV6/04 | **M2** ^a^ |  | DQ493177 | Ck/Kulon_Progo/BBVW/05 |
| DQ492918 | Ck/Mangarai_NTT/BPPV6/04 | AY651407 | A/Ck/HK/YU324/2003 | DQ493173 | Ck/Purworejo/BBVW/05 |
| DQ492920 | Ck/Kupang_2_NTT/BPPV6/04 | AY651374 | Ck/IDN/BL/03 | DQ493168 | Ck/Magetan/BBVW/05 |
| DQ492921 | Ck/Kupang_3_NTT/BPPV6/04 | AY651376 | Ck/IDN/PA/03 | DQ321195 | Ck/Bantul/BBVet_I/05 |
| DQ492922 | Ck/Kupang_1_NTT/BPPV6/04 | AY651377 | Ck/IDN/2A/03 | DQ321196 | Ck/Wajo/BBVM/05 |
| DQ492923 | Ck/Pangkalpinang/BPPV3/04 | DQ320995 | Ck/Wonosobo/BPPV4/03 | DQ321191 | Ck/Salatiga/BBVet_I/05 |
| DQ492924 | Tk/Kedaton/BPPV3/04 | DQ492906 | Ck/Pekalongan/BPPV4/03 | CY014174 | IDN/5/05 |
| DQ492910 | Qa/Yogjakarta/BBVet_IX/04 | DQ492907 | Ck/Sragen/BPPV4/03 | CY014181 | IDN/CDC7/05 |
| DQ492930 | Ck/Kulon_Progo/BBVet_XII_2/04 | EF473084 | Ck/IDN/11/03 | CY014189 | Ck/IDN/CDC25/05 |
| DQ320996 | Ck/Yogjakarta/BBVet_IX/04 | EF473079 | Ck/IDN/7/03 | CY014196 | Ck/IDN/CDC24/05 |
| DQ320997 | Ck/Kulon_Progo/BBVet_XII_1/04 | AY651378 | Ck/IDN/4/04 | CY014268 | IDN/CDC194P/05 |
| DQ492905 | Ck/Ngawi/BPPV4/04 | AY651379 | Ck/IDN/5/04 | CY014255 | IDN/CDC184/05 |
| DQ492903 | Ck/Malang/BBVet_IV/04 | AY651375 | Dk/IDN/MS/04 | CY014267 | IDN/CDC287E/05 |
| DQ492904 | Ck/Magetan/BBVW/05 | DQ492908 | Qa/Boyolali/BPPV4/04 | CY014265 | IDN/CDC292T/05 |
| DQ320998 | Ck/Bantul/BBVet_I/05 | DQ492913 | Ck/Purwakarta/BBVet_IV/04 | CY014547 | IDN/CDC759/06 |
| DQ320999 | Ck/Wajo/BBVM/05 | DQ492914 | Qa/Tasikmalaya/BPPV4/04 | CY014264 | IDN/CDC329/06 |
| DQ320994 | Ck/Salatiga/BBVet_I/05 | DQ492915 | Ck/Bangli_Bali/BPPV6_2/04 | CY014266 | IDN/CDC326/06 |
| DQ492911 | Ck/Gunung_Kidal/BBVW/05 | DQ492916 | Ck/Bangli_Bali/BBPV6_1/04 | CY014260 | Ct/IDN/CDC1/06 |
| DQ492912 | Ck/Kulon_Progo/BBVW/05 | DQ492917 | Ck/Jembrana/BPPV6/04 | CY014259 | IDN/CDC357/06 |
| DQ492925 | Ck/Simalanggang/BPPVI/05 | DQ492918 | Ck/Mangarai_NTT/BPPV6/04 | CY014261 | IDN/CDC370/06 |
| DQ492926 | Ck/Tebing_Tinggi/BPPVI/05 | DQ492920 | Ck/Kupang_2_NTT/BPPV6/04 | CY014263 | IDN/CDC390/06 |
| DQ492927 | Ck/Dairi/BPPVI/05 | DQ492921 | Ck/Kupang_3_NTT/BPPV6/04 | CY014315 | IDN/CDC523/06 |
| DQ492928 | Ck/Deli_Serdang/BPPVI/05 | DQ492922 | Ck/Kupang_1_NTT/BPPV6/04 | CY014388 | IDN/CDC582/06 |
| DQ492929 | Ck/Tarutung/BPPVI/05 | DQ492923 | Ck/Pangkalpinang/BPPV3/04 | CY014300 | IDN/CDC597/06 |
| DQ492919 | Dk/Parepare/BBVM/05 | DQ492924 | Tk/Kedaton/BPPV3/04 | CY014292 | IDN/CDC596/06 |
| DQ492909 | Ck/Purworejo/BBVW/05 | DQ492910 | Qa/Yogjakarta/BBVet_IX/04 | CY014307 | IDN/CDC599/06 |
| CY014173 | IDN/5/05 | DQ492930 | Ck/Kulon_Progo/BBVet_XII_2/04 | CY014400 | IDN/CDC610/06 |
| CY014180 | IDN/CDC7/05 | DQ320996 | Ck/Yogjakarta/BBVet_IX/04 | CY014408 | IDN/CDC623/06 |
| CY014195 | Ck/IDN/CDC24/05 | DQ320997 | Ck/Kulon_Progo/BBVet_XII_1/04 | CY014424 | IDN/CDC624/06 |
| CY014188 | Ck/IDN/CDC25/05 | DQ492905 | Ck/Ngawi/BPPV4/04 | CY014440 | IDN/CDC625/06 |
| CY014225 | IDN/CDC194P/05 | DQ492903 | Ck/Malang/BBVet_IV/04 | CY014448 | IDN/CDC634/06 |
| CY014214 | IDN/CDC184/05 | DQ492904 | Ck/Magetan/BBVW/05 | CY014517 | IDN/CDC644/06 |
| CY014226 | IDN/CDC287E/05 | DQ320998 | Ck/Bantul/BBVet_I/05 | CY014284 | IDN/CDC595/06 |
| CY014215 | IDN/CDC292T/05 | DQ320999 | Ck/Wajo/BBVM/05 | CY014276 | IDN/CDC594/06 |
| CY014546 | IDN/CDC759/06 | DQ320994 | Ck/Salatiga/BBVet_I/05 | CY014488 | IDN/CDC669/06 |
| CY014219 | IDN/CDC329/06 | DQ492911 | Ck/Gunung_Kidal/BBVW/05 | CY014504 | IDN/CDC699/06 |
| CY014216 | IDN/CDC326/06 | DQ492912 | Ck/Kulon_Progo/BBVW/05 | CY014533 | IDN/CDC739/06 |
| CY014221 | Ct/IDN/CDC1/06 | DQ492925 | Ck/Simalanggang/BPPVI/05 | CY014541 | IDN/CDC742/06 |
| CY014220 | IDN/CDC357/06 | DQ492926 | Ck/Tebing_Tinggi/BPPVI/05 | CY017666 | IDN/CDC835/06 |
| CY014222 | IDN/CDC370/06 | DQ492927 | Ck/Dairi/BPPVI/05 | CY017674 | IDN/CDC836/06 |
| CY014224 | IDN/CDC390/06 | DQ492928 | Ck/Deli_Serdang/BPPVI/05 | CY017692 | IDN/CDC887/06 |
| CY014314 | IDN/CDC523/06 | DQ492929 | Ck/Tarutung/BPPVI/05 | CY017642 | IDN/CDC938/06 |
| CY014385 | IDN/CDC582/06 | DQ492919 | Dk/Parepare/BBVM/05 | CY017658 | IDN/CDC940/06 |
| CY014299 | IDN/CDC597/06 | DQ492909 | Ck/Purworejo/BBVW/05 | CY019412 | IDN/CDC1046/07 |
| CY014291 | IDN/CDC596/06 | CY014173 | IDN/5/05 | CY019428 | IDN/CDC1047/07 |
| CY014306 | IDN/CDC599/06 | CY014180 | IDN/CDC7/05 | CY019356 | IDN/CDC1031/07 |
| CY014397 | IDN/CDC610/06 | CY014195 | Ck/IDN/CDC24/05 | CY019388 | IDN/CDC1032/07 |
| CY014405 | IDN/CDC623/06 | CY014188 | Ck/IDN/CDC25/05 | ISDN136845 | IDN/298H/06 |
| CY014421 | IDN/CDC624/06 | CY014225 | IDN/CDC194P/05 | ISDN128030 | IDN/6/05 |
| CY014437 | IDN/CDC625/06 | CY014214 | IDN/CDC184/05 | ISDN130916 | IDN/195H/05 |
| CY014445 | IDN/CDC634/06 | CY014226 | IDN/CDC287E/05 |  |  |
| CY014514 | IDN/CDC644/06 | CY014215 | IDN/CDC292T/05 |  |  |
| CY014283 | IDN/CDC595/06 | CY014546 | IDN/CDC759/06 |  |  |
| CY014275 | IDN/CDC594/06 | CY014219 | IDN/CDC329/06 |  |  |
| CY014485 | IDN/CDC669/06 | CY014216 | IDN/CDC326/06 |  |  |
| CY014501 | IDN/CDC699/06 | CY014221 | Ct/IDN/CDC1/06 |  |  |
| CY014532 | IDN/CDC739/06 | CY014220 | IDN/CDC357/06 |  |  |
| CY017665 | IDN/CDC835/06 | CY014222 | IDN/CDC370/06 |  |  |
| CY017673 | IDN/CDC836/06 | CY014224 | IDN/CDC390/06 |  |  |
| CY017691 | IDN/CDC887/06 | CY014314 | IDN/CDC523/06 |  |  |
| CY017641 | IDN/CDC938/06 | CY014385 | IDN/CDC582/06 |  |  |
| CY017657 | IDN/CDC940/06 | CY014299 | IDN/CDC597/06 |  |  |
| CY019411 | IDN/CDC1046/07 | CY014291 | IDN/CDC596/06 |  |  |
| CY019427 | IDN/CDC1047/07 | CY014306 | IDN/CDC599/06 |  |  |
| CY019355 | IDN/CDC1031/07 | CY014397 | IDN/CDC610/06 |  |  |
| CY019387 | IDN/CDC1032/07 | CY014405 | IDN/CDC623/06 |  |  |
| ISDN136842 | IDN/298H/06 | CY014421 | IDN/CDC624/06 |  |  |
| ISDN128028 | IDN/6/05 | CY014437 | IDN/CDC625/06 |  |  |
| **NS1** ^a^ |  | CY014445 | IDN/CDC634/06 |  |  |
| AY651572 | A/Ck/HK/YU324/2003 | CY014514 | IDN/CDC644/06 |  |  |
| AY651539 | Ck/IDN/BL/03 | CY014283 | IDN/CDC595/06 |  |  |
| AY651541 | Ck/IDN/PA/03 | CY014275 | IDN/CDC594/06 |  |  |
| AY651543 | Ck/IDN/2A/03 | CY014485 | IDN/CDC669/06 |  |  |
| DQ493170 | Ck/Pekalongan/BPPV4/03 | CY014501 | IDN/CDC699/06 |  |  |
| DQ493171 | Ck/Sragen/BPPV4/03 | CY014532 | IDN/CDC739/06 |  |  |
| DQ321192 | Ck/Wonosobo/BPPV4/03 | CY017665 | IDN/CDC835/06 |  |  |
| AY651544 | Ck/IDN/5/04 | CY017673 | IDN/CDC836/06 |  |  |
| AY651542 | Ck/IDN/4/04 | CY017691 | IDN/CDC887/06 |  |  |
| AY651540 | Dk/IDN/MS/04 | CY017641 | IDN/CDC938/06 |  |  |
| DQ321193 | Ck/Yogjakarta/BBVet_IX/04 | CY017657 | IDN/CDC940/06 |  |  |
| DQ321194 | Ck/Kulon_Progo/BBVet_XII_1/04 | CY019411 | IDN/CDC1046/07 |  |  |
| DQ493167 | Ck/Malang/BBVet_IV/04 | CY019427 | IDN/CDC1047/07 |  |  |
| DQ493172 | Qa/Boyolali/BPPV4/04 | CY019355 | IDN/CDC1031/07 |  |  |
| DQ493174 | Qa/Yogjakarta/BBVet_IX/04 | CY019387 | IDN/CDC1032/07 |  |  |
| DQ493175 | Ck/Kulon_Progo/BBVet_XII_2/04 | ISDN136842 | IDN/298H/06 |  |  |
| DQ493185 | Ck/Kupang_2_NTT/BPPV6/04 | ISDN128028 | IDN/6/05 |  |  |
| DQ493186 | Ck/Kupang_3_NTT/BPPV6/04 | **NS2** ^a^ |  |  |  |
| DQ493187 | Ck/Kupang_1_NTT/BPPV6/04 | AY651572 | A/Ck/HK/YU324/2003 |  |  |
| DQ493188 | Ck/Pangkalpinang/BPPV3/04 | AY651539 | Ck/IDN/BL/03 |  |  |
| DQ493189 | Tk/Kedaton/BPPV3/04 | AY651541 | Ck/IDN/PA/03 |  |  |
| DQ493178 | Ck/Purwakarta/BBVet_IV/04 | AY651543 | Ck/IDN/2A/03 |  |  |
| DQ493179 | Qa/Tasikmalaya/BPPV4/04 | DQ493170 | Ck/Pekalongan/BPPV4/03 |  |  |
| DQ493180 | Ck/Bangli_Bali/BBPV6_1/04 | DQ493171 | Ck/Sragen/BPPV4/03 |  |  |
| DQ493181 | Ck/Bangli_Bali/BPPV6_2/04 | DQ321192 | Ck/Wonosobo/BPPV4/03 |  |  |
| DQ493182 | Ck/Jembrana/BPPV6/04 | AY651544 | Ck/IDN/5/04 |  |  |
| DQ493183 | Ck/Mangarai_NTT/BPPV6/04 | AY651542 | Ck/IDN/4/04 |  |  |
| DQ493169 | Ck/Ngawi/BPPV4/04 | AY651540 | Dk/IDN/MS/04 |  |  |
| DQ493184 | Dk/Parepare/BBVM/05 | DQ321193 | Ck/Yogjakarta/BBVet_IX/04 |  |  |
| DQ493190 | Ck/Simalanggang/BPPVI/05 | DQ321194 | Ck/Kulon_Progo/BBVet_XII_1/04 |  |  |
| DQ493191 | Ck/Tebing_Tinggi/BPPVI/05 | DQ493167 | Ck/Malang/BBVet_IV/04 |  |  |
| DQ493192 | Ck/Dairi/BPPVI/05 | DQ493172 | Qa/Boyolali/BPPV4/04 |  |  |
| DQ493193 | Ck/Deli_Serdang/BPPVI/05 | DQ493174 | Qa/Yogjakarta/BBVet_IX/04 |  |  |
| DQ493194 | Ck/Tarutung/BPPVI/05 | DQ493175 | Ck/Kulon_Progo/BBVet_XII_2/04 |  |  |
| DQ493176 | Ck/Gunung_Kidal/BBVW/05 | DQ493185 | Ck/Kupang_2_NTT/BPPV6/04 |  |  |
| DQ493177 | Ck/Kulon_Progo/BBVW/05 | DQ493186 | Ck/Kupang_3_NTT/BPPV6/04 |  |  |
| DQ493173 | Ck/Purworejo/BBVW/05 | DQ493187 | Ck/Kupang_1_NTT/BPPV6/04 |  |  |
| DQ493168 | Ck/Magetan/BBVW/05 | DQ493188 | Ck/Pangkalpinang/BPPV3/04 |  |  |
| DQ321195 | Ck/Bantul/BBVet_I/05 | DQ493189 | Tk/Kedaton/BPPV3/04 |  |  |
| DQ321196 | Ck/Wajo/BBVM/05 | DQ493178 | Ck/Purwakarta/BBVet_IV/04 |  |  |
| DQ321191 | Ck/Salatiga/BBVet_I/05 | DQ493180 | Ck/Bangli_Bali/BBPV6_1/04 |  |  |
| CY014174 | IDN/5/05 | DQ493181 | Ck/Bangli_Bali/BPPV6_2/04 |  |  |
| CY014181 | IDN/CDC7/05 | DQ493182 | Ck/Jembrana/BPPV6/04 |  |  |
| CY014189 | Ck/IDN/CDC25/05 | DQ493183 | Ck/Mangarai_NTT/BPPV6/04 |  |  |
| CY014196 | Ck/IDN/CDC24/05 | DQ493169 | Ck/Ngawi/BPPV4/04 |  |  |
| CY014268 | IDN/CDC194P/05 | DQ493184 | Dk/Parepare/BBVM/05 |  |  |
| CY014255 | IDN/CDC184/05 | DQ493190 | Ck/Simalanggang/BPPVI/05 |  |  |
| CY014267 | IDN/CDC287E/05 | DQ493191 | Ck/Tebing_Tinggi/BPPVI/05 |  |  |
| CY014265 | IDN/CDC292T/05 | DQ493192 | Ck/Dairi/BPPVI/05 |  |  |
| CY014547 | IDN/CDC759/06 | DQ493193 | Ck/Deli_Serdang/BPPVI/05 |  |  |
| CY014264 | IDN/CDC329/06 | DQ493194 | Ck/Tarutung/BPPVI/05 |  |  |
| CY014266 | IDN/CDC326/06 | DQ493176 | Ck/Gunung_Kidal/BBVW/05 |  |  |
| CY014260 | Ct/IDN/CDC1/06 | DQ493177 | Ck/Kulon_Progo/BBVW/05 |  |  |
| CY014259 | IDN/CDC357/06 | DQ493173 | Ck/Purworejo/BBVW/05 |  |  |
| CY014261 | IDN/CDC370/06 | DQ493168 | Ck/Magetan/BBVW/05 |  |  |
| CY014263 | IDN/CDC390/06 | DQ321195 | Ck/Bantul/BBVet_I/05 |  |  |
| CY014315 | IDN/CDC523/06 | DQ321196 | Ck/Wajo/BBVM/05 |  |  |
| CY014388 | IDN/CDC582/06 | DQ321191 | Ck/Salatiga/BBVet_I/05 |  |  |
| CY014300 | IDN/CDC597/06 | CY014174 | IDN/5/05 |  |  |
| CY014292 | IDN/CDC596/06 | CY014181 | IDN/CDC7/05 |  |  |
| CY014307 | IDN/CDC599/06 | CY014189 | Ck/IDN/CDC25/05 |  |  |
| CY014400 | IDN/CDC610/06 | CY014196 | Ck/IDN/CDC24/05 |  |  |
| CY014408 | IDN/CDC623/06 | CY014268 | IDN/CDC194P/05 |  |  |
| CY014424 | IDN/CDC624/06 | CY014255 | IDN/CDC184/05 |  |  |
| CY014440 | IDN/CDC625/06 | CY014267 | IDN/CDC287E/05 |  |  |
| CY014448 | IDN/CDC634/06 | CY014265 | IDN/CDC292T/05 |  |  |
| CY014517 | IDN/CDC644/06 | CY014547 | IDN/CDC759/06 |  |  |
| CY014284 | IDN/CDC595/06 | CY014264 | IDN/CDC329/06 |  |  |
| CY014276 | IDN/CDC594/06 | CY014266 | IDN/CDC326/06 |  |  |
| CY014488 | IDN/CDC669/06 | CY014260 | Ct/IDN/CDC1/06 |  |  |
| CY014504 | IDN/CDC699/06 | CY014259 | IDN/CDC357/06 |  |  |
| CY014533 | IDN/CDC739/06 | CY014261 | IDN/CDC370/06 |  |  |
| CY014541 | IDN/CDC742/06 | CY014263 | IDN/CDC390/06 |  |  |
| CY017666 | IDN/CDC835/06 | CY014315 | IDN/CDC523/06 |  |  |
| CY017674 | IDN/CDC836/06 | CY014388 | IDN/CDC582/06 |  |  |
| CY017692 | IDN/CDC887/06 | CY014300 | IDN/CDC597/06 |  |  |
| CY017642 | IDN/CDC938/06 | CY014292 | IDN/CDC596/06 |  |  |
| CY017658 | IDN/CDC940/06 | CY014307 | IDN/CDC599/06 |  |  |
| CY019412 | IDN/CDC1046/07 | CY014400 | IDN/CDC610/06 |  |  |
| CY019428 | IDN/CDC1047/07 | CY014408 | IDN/CDC623/06 |  |  |
| CY019356 | IDN/CDC1031/07 | CY014424 | IDN/CDC624/06 |  |  |
| CY019388 | IDN/CDC1032/07 | CY014440 | IDN/CDC625/06 |  |  |
| ISDN136845 | IDN/298H/06 | CY014448 | IDN/CDC634/06 |  |  |
| ISDN128030 | IDN/6/05 | CY014517 | IDN/CDC644/06 |  |  |
| ISDN130916 | IDN/195H/05 | CY014284 | IDN/CDC595/06 |  |  |
|  |  | CY014276 | IDN/CDC594/06 |  |  |
|  |  | CY014488 | IDN/CDC669/06 |  |  |
|  |  | CY014504 | IDN/CDC699/06 |  |  |
|  |  | CY014533 | IDN/CDC739/06 |  |  |
|  |  | CY014541 | IDN/CDC742/06 |  |  |
|  |  | CY017666 | IDN/CDC835/06 |  |  |
|  |  | CY017674 | IDN/CDC836/06 |  |  |
|  |  | CY017692 | IDN/CDC887/06 |  |  |
|  |  | CY017642 | IDN/CDC938/06 |  |  |
|  |  | CY017658 | IDN/CDC940/06 |  |  |
|  |  | CY019412 | IDN/CDC1046/07 |  |  |
|  |  | CY019428 | IDN/CDC1047/07 |  |  |
|  |  | CY019356 | IDN/CDC1031/07 |  |  |
|  |  | CY019388 | IDN/CDC1032/07 |  |  |
|  |  | ISDN136845 | IDN/298H/06 |  |  |
|  |  | ISDN128030 | IDN/6/05 |  |  |
|  |  | ISDN130916 | IDN/195H/05 |  |  |

^a^ The regions of nucleotide sequences of the alignments used for building phylogenetic trees are described in following (reference sequences are in parentheses):

PB2: 1008nt-2270nt (AY651706);

PB1: 70nt-1461nt (AY651652);

PA: 1394nt-2140nt (AY651600);

HA: 55nt-1665nt (EU124083, 1033nt-1038nt removed because of gaps);

NP: 1nt-868nt (AY651486);

NA: 43nt-1037nt (AY651434);

MP: 1nt-930nt (AY651375);

M1: 1nt-756nt (AY651375);

M2: 1nt-26nt, 715nt-928nt (AY651375);

NS: 45nt-768nt (AY651540);

NS1: 45nt-680nt (AY651540);

NS2: 493nt-801nt (AY651540)

Note that all position indexes are inclusive. Alignments used for detecting positively selected sites are nearly full-length sequences.

**End of Table S7**
